# Hardware and software design trends and considerations in gamified stroke rehabilitation: Results from a systematic review

**DOI:** 10.1007/s11517-026-03518-y

**Published:** 2026-03-03

**Authors:** Juan J. Sánchez-Gil, Aurora Sáez Manzano, Juan José Ochoa-Sepúlveda, Laura Muñoz-Millán, David Cáceres-Gómez, Rafael López-Luque, Eduardo Cañete-Carmona

**Affiliations:** 1 Departamento de Ingeniería Electrónica y de Computadores, Universidad de Córdoba, Edificio Leonardo Da Vinci, Campus de Rabanales, Córdoba, 14071 España; 2 Instituto de Neurociencias, Hospital Cruz Roja, P.º de la Victoria, Córdoba, 14004 España; 3 Departamento de Ciencia de la Computación e Inteligencia Artificial, Universidad de Córdoba, Edificio Marie Curie, Campus de Rabanales, Córdoba, 14071 España

**Keywords:** Stroke, Gamification, Gamified design, Hardware, Software, Design considerations

## Abstract

Gamification in stroke rehabilitation is a method that applies game-design elements to enhance patient engagement, motivation, and functional recovery. This study reviews 68 gamified prototypes for post-stroke neurorehabilitation published between 2019 and 2023, integrating hardware, software, and gamification design criteria under a unified framework. Technological trends, therapeutic domains, and gamification components are analyzed, yielding eight overarching guidelines for developing patient-centered devices. Results confirm a predominance of solutions targeting the functional domain (79% motor), an expansion of immersive environments (VR/IVR, 66%), and the emergence of hybrid systems that combine robotics, sensors, and artificial intelligence algorithms to adapt assistance in real time. However, affective and social domains remain underrepresented, and clinical interoperability continues to pose challenges. Based on current design criteria, future directions propose advanced gamification frameworks that foster intrinsic motivation. By laying the groundwork for more motivating, personalized, and cost-effective gamified therapies, this work offers a practical guide for engineers, therapists, and developers.

**Graphical abstract:**

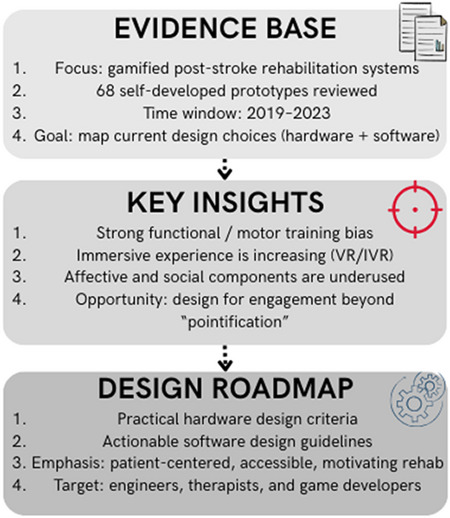

**Supplementary Information:**

The online version contains supplementary material available at 10.1007/s11517-026-03518-y.

## Introduction

Gamification, as seen in the literature, consists of the intro- duction of game elements into non-game environments [1–4]. It has several intrinsic aspects and characteristics that are advantageous in different contexts, mainly to promote increased motivation or engagement [5, 6]. Considering these possibilities offered by gamification, the effects of its integration in different environments have been evaluated in the literature for several years. Among them are, for example, the educational context [7, 8], talent acquisition [9], the elderly [10], mental health [11, 12], and other health contexts such as cancer [13] and neurological pathologies such as stroke rehabilitation [14, 15].

With regard to stroke, it is a cerebrovascular accident caused by the obstruction of the blood vessels of the brain (ischemic stroke) or by their rupture (hemorrhagic stroke) [16–18]. Stroke can leave a number of sequels. Among them, one third of patients who suffer a stroke usually present a disability after the episode [19, 20], while they can also induce episodes of anxiety and/or depression [21, 22], among others.

Considering the bleak outlook of this pathology and the lack of motivation and engagement present in patients after an stroke episode [23, 24], ensuring sufficient and necessary rehabilitation is crucial for the physical and cognitive recovery [25].

**Fig. 1:**
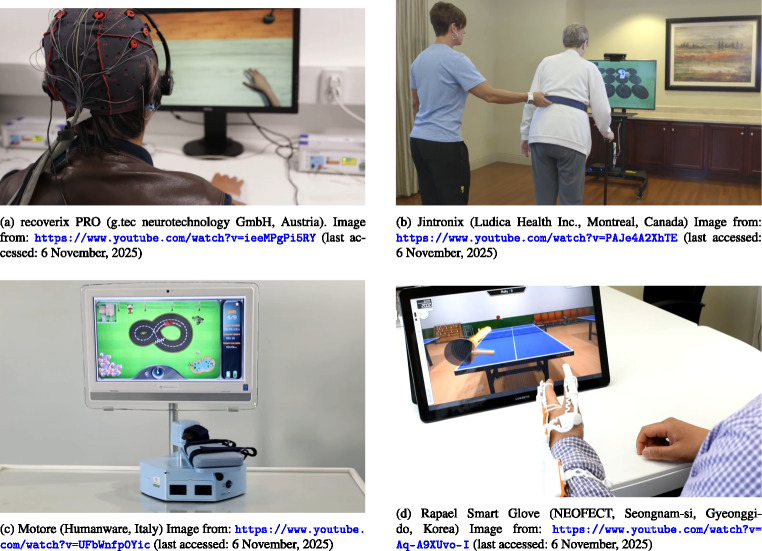
Presentation of representative gamified technologies for stroke rehabilitation: (**a**) tDCS device integrated with Virtual Reality (VR) environments; (**b**) depth camera with VR games; (**c**) motorized platform, and (**d**) instrumented glove, both with Serious Virtual Games (SVG)

In this context, gamification has emerged as a promis- ing strategy to enhance motivation and engagement in post- stroke rehabilitation. Figure 1 shows some examples of how Serious Games (SG) have been integrated into different stroke rehabilitation therapies.

However, current implementations lack standardization and often neglect non-functional patient domains. This work builds upon a prior systematic review [16] to analyze recent gamified systems, with a focus on their hardware, software, and design principles.

While the aforementioned systematic review synthesized the clinical and patient-reported effects of gamified interventions (motivation, usability, emotional and social outcomes), the present study conducts a new technical and design-oriented analysis of the previously reviewed technologies. Specifically, the focus is on the subset of non-commercial, self-developed devices, which are examined through a framework that distinguishes between patient domains (affective, functional, social) and therapy settings (technology, game environment, implementation). Based on this, a detailed codification of the characteristics of the hardware, software, and gamification is created. As a result, design criteria are extracted to be used in future gamified stroke rehabilitation systems.

After a brief introduction to gamification, neurorehabilitation and stroke in this chapter, Sect. 2 presents the background of this work, together with the proposal made and the objectives pursued. Then, Sect. 4 presents some key con- cepts to properly understand gamification. Then, in Sect. 5 the analyzed statistics of the gamified systems used in the last years are shown, as a result of the systematic review carried out in [16]. Then, in Sect. 6 based on what is found in the analysis, some key design considerations found in the literature are detailed. In Sect. 7, after a thorough analysisof current trends and a review of design considerations, future directions for gamified neurorehabilitation systems are detailed. The results of this work are discussed in Sect. 8 and conclusions are presented in Sect. 9.

## Related work

This section provides the background to the work presented here. The literature on the gamification of stroke rehabili- tation processes is extensive and is attracting increasing in- terest in the scientific community. There are many works that attempt to define what gamification is, how gamified systems are designed, or what results this methodology provides in the field of neurorehabilitation.

In [26] the authors review commercial hand rehabilita- tion systems for stroke patients. They provide a sort of catalog of the devices they find, making a valuable contribution to a better understanding of these technologies. They divide them into two main groups: those that involve contact (robots, gloves, etc.) and those that do not (Virtual Reality (VR) technologies). They point out that the heterogeneity of the studies makes direct comparisons difficult, in addition to the limitation of the lack of specific game protocols for hand rehabilitation.

On the other hand, in [27], the authors review the current status and barriers that patients face in accessing rehabilitation based on mHealth technologies. They highlight some problems at the infrastructure level of these technologies, especially at the software level (unintuitive interfaces), network connectivity, and hardware device failures. They also highlight the lack of support systems, which affects motivation.

In [28] they analyze assistive technology for upper limb rehabilitation of stroke patients. They grouped the findings into five major themes: (1) promoting arm and hand performance, (2) obtaining patient attitudes of familiarity and motivation toward the technology, (3) facilitating decision- making processes based on scientific evidence, cost, and trust of healthcare professionals, (4) system usability, and (5) applicability in practice. This work highlights the complex web of factors involved in the adoption of assistive technology in upper extremity rehabilitation of stroke patients.

In [29] they deal exhaustively with automated systems also for rehabilitation of the upper limb of stroke patients. They make a comparative work of the types of sensors found in these systems: accelerometers, Inertial Measurement Unit (IMU), bending sensors, pressure sensors, among others. They analyze the evolution of research from manual assessment with clinical scales to automated methods.

Then, in [30], they perform a scoping review to identify concepts and topics in information technology research for stroke neurorehabilitation. The topics were grouped into four: (1) robotics (motorized devices), (2) software (machine learning algorithms and sensor signal processing), (3) functional (electrical or brain stimulation interventions), and (4) cognitive (gamified environments to work on cognitive skills).

In [31] they perform a scoping review of the require- ments necessary for the design of a home-based robotic upper extremity rehabilitation system. They identify four global requirements from which they develop a list of more specific criteria for robotic technology: (1) functionality (11 requirements), (2) usability (16 requirements), (3) software (14 requirements), and (4) safety (1 requirement).

In [6] they review trends in design features for gamified systems for use in telerehabilitation. In [14] they analyze the challenges and benefits of motor recovery after stroke. They group gamified technologies and systems into three categories: (1) non-robotic (mainly commercial such as Kinect, Nintendo Wii, etc.), (2) robotic, and (3) Inmersive Virtual Reality (IVR) (immersing the patient in realistic environments).

Finally, in [32] they offer trends in data mining techniques for the generation of predictive models in recovery and in exercise recommendation systems. In addition, they offer gamification usage maps, highlighting exergames, SG and VR. Finally, they identify some gaps such as poor validation of models and limited development of intelligent systems.

### Proposal

This section will now explain what this work contributes in relation to the current literature reviewed. First, it is worth noting the primary focus on gamification in this work, as well as in [6, 14, 26, 32]. In [28] it is treated at a secondary level (mentioned, and somewhat detailed, briefly). In [27] it occurs at a more tertiary level (gamification is mentioned very casually, as an existing possibility within the systems they review). Finally, in [29, 30] the treatment of gamification is null. In this proposed work, the treatment of gamification, as already mentioned, is primary. It is not enough to have a good understanding of the mechanical systems themselves and their design, but a proper correlation of these with respect to the gamified methodology.

In fact, as pointed out in [32, 33], there is a lack of in- depth studies on the effectiveness of gamified components, in understanding integration into healthcare systems and understanding game mechanics and design elements. This is a gap that needs to be taken into account, as generally the approaches that have been examined for gamification tend to be in very specific areas, such as in relation to telerehabilitation [6, 34], current challenges and more general issues [14, 32, 35, 36], or upper extremity rehabilitation [26, 31, 37–39]. This lack of in-depth studies regarding gamified components is intended to be effectively addressed in the work proposed here.

Such an assiduous focus on upper extremity rehabili- tation alone prevents a more complete analysis of all do- mains within stroke rehabilitation [16, 30]. This highlights the need for the proposal made here, as the proposed analysis is intended to cover more complete domains of gamified stroke neurorehabilitation, as will be discussed below.

The literature on the most optimal considerations for rehabilitation systems has already addressed issues such as optimizing technologies to make them more economical and accessible. For example, this line is reviewed in [40], where challenges and recommendations for robotic technology are discussed. In the proposal made here, the picture is broader: not only isolated robotic technology is discussed. In addition, all current systems are analyzed and recommendations and design criteria are proposed for each type of technology. 

In short, as far as the authors are aware, there is no analysis of the software and hardware of current gamified stroke systems together with design criteria that also fully integrate gamification concepts based on a broad and detailed analysis of these gamified systems. This would summarize the issue highlighted in [41], where the authors comment on the need to investigate gamified environments in robotic devices for wrist rehabilitation to increase patient motivation and adherence. However, as mentioned above, the scope of this work is opened up in order to make it more comprehensive and complete. In this way, it also aims to contribute to a deeper understanding of gamified technologies for stroke neurorehabilitation on a technical and theoretical level.

### Objectives of this work

The analysis of the newly designed systems, along with the reviewed design criteria, has the following objectives:

- **O1. To review** the current hardware and software used in stroke rehabilitation and their subsequent integration into gamified systems.
- **O2. To analyze** how gamification is optimally integrated in recent systems by carrying out an exhaustive analysis of the reviewed works.
- **O3.** Although other works have gone into more detail on more specific issues (sensors, treadmills, deep learning, robots, among others), this work tries **to synthesize** the most relevant design considerations and their optimal integration in relation to gamification.

With this, a broader catalog of currently designed gamified systems (2019 to 2023) is provided, along with a synthesis that helps to understand more fully the gamified systems in neurorehabilitation and their integral design, taking into account not only the hardware but also the software and the considerations related to gamification holistically.

## Summary of the previous systematic review

The present work builds on a previous systematic review on gamified devices for stroke rehabilitation [16]. That study analyzed the emotional, social, personal and functional effects of gamification in stroke patients. It was conducted according to the PRISMA 2020 guidelines. This section briefly summarize the main methodological aspects and key findings from [16] that are relevant for the present study.

### Search strategy and selection criteria

As already said, the systematic review followed PRISMA 2020 and included a comprehensive search in five databases: IEEE Xplore, PubMed, Springer Link, APA PsycInfo and ScienceDirect. Authors in [16] considered empirical studies published between January 2019 and December 2023.

The search strategy was:

- Serious games **AND** stroke rehabilitation.
- Gamified devices **AND** stroke rehabilitation.
- Virtual reality **AND** stroke rehabilitation.
- Robotic **AND** stroke rehabilitation.
- Electrical stimulation **AND** stroke rehabilitation.

On the other hand, Inclusion Criteria (IC) were: (IC1) publications between 2019 and 2023; (IC2) written in English; (IC3) JCR-indexed journal articles; (IC4) empirical (non-review) studies; (IC5) conducted in stroke patients (sub-acute or chronic); and (IC6) quantifying the effects of gamified rehabilitation in terms of usability, motivation, engagement or other outcomes related to patients’ emotional, social or personal responses to the gamified therapy.

Exclusion Criteria (EC1-6) were: studies older than 2019, non-English, non-indexed, or non-empirical articles, studies not involving stroke patients, or studies that did not quantify the effects of gamification on the patients’ experience.

Most studies involving neurological patients reported approval by a local ethics committee and written informed consent from participants, as indicated in the original articles. However, this information was not systematically reported in all publications, nor was it used as an inclusion criterion in the review on which this work is based. Since the objective of this study is to extract design criteria from gamified systems, not reevaluate the ethics of the original clinical trials, this information is mentioned solely for contextualization purposes.

**Fig. 2:**
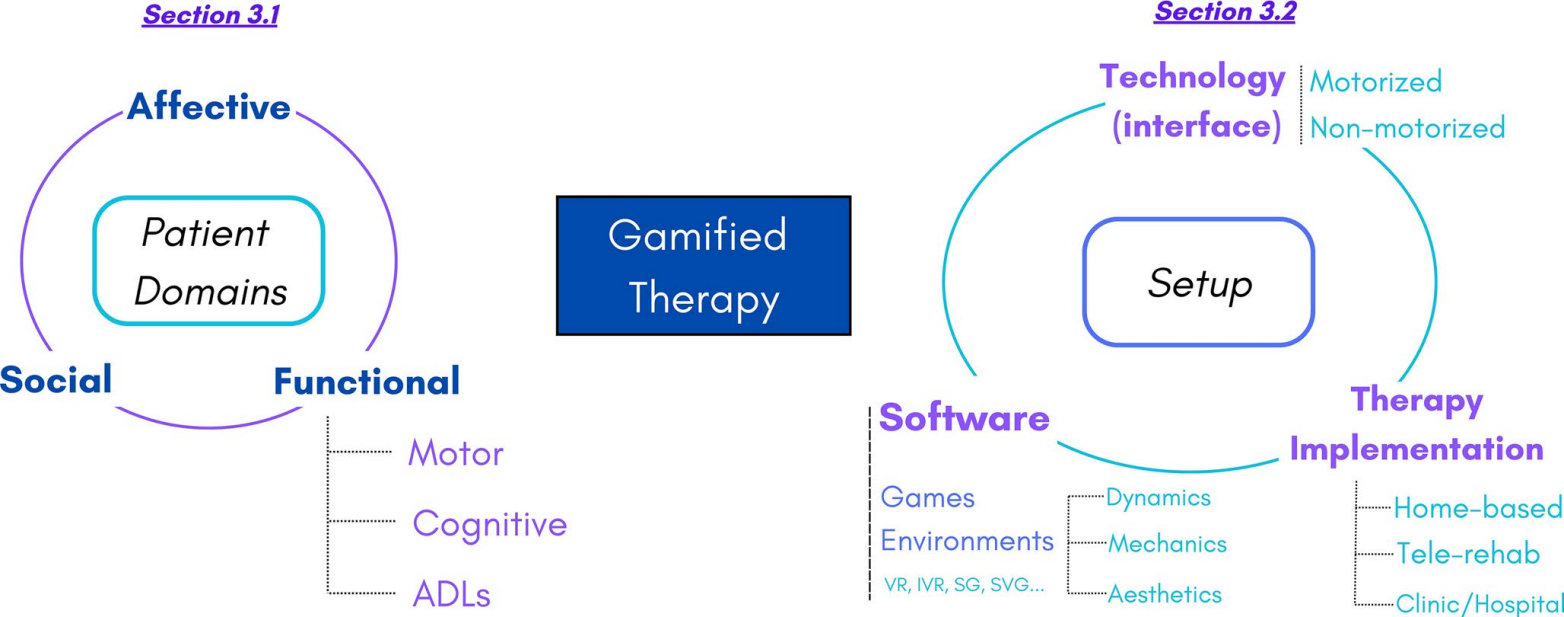
Flowchart for selecting literature based on the PRISMA 2020 guidelines

Following the PRISMA flow (Fig. 2), the initial search identified 59.286 records. After filtering by years, language, indexation and study type, 6.421 articles remained. Then, authors removed 3.900 duplicates and screened 2.521 works. Of these, 1.279 were excluded because they were not related to stroke, and 1.169 did not report quantitative or qualitative measures of the effects of gamified therapy on emotional, social or personal domains. Finally, 169 studies met all the criteria and were included in that review.

### Study characteristics

The 169 included studies (comprising a total of 6.404 stroke patients) were grouped into four main technological categories, based mainly in [14]: (1) robotic and motorized devices, (2) non-motorized devices based on serious games or serious virtual games, (3) virtual and augmented reality solutions (VR, Augmented Reality (AR) and IVR), and. (4) Neuromuscular Electrical Stimulation (NMES) systems. This classification allowed to compare how gamification is implemented across different rehabilitation technologies.

Overall, 62% of patients suffered an ischemic stroke and 16% a hemorrhagic stroke, whereas 22% were not specified. Regarding time since stroke, 43% of patients were in the subacute phase (less than 6 months since stroke), 39% in the chronic phase (more than 6 months since stroke) and 18% not specified. On the other hand, 61% were male, 38% were female, and 1% were unspecified. Most samples consisted of middle-aged and older adults, typically between 50 and 75 years, as reported in the individual studies. Most interventions targeted upper-limb motor function, although lower-limb rehabilitation and mixed protocols were also analyzed. 

Across studies, gamified devices were generally associated with improvements in motor and/or cognitive outcomes, but also with high levels of patient satisfaction, motivation and acceptance of therapy. A wide variety of clinical scales and patient-reported tests were used to assess usability, engagement, emotional response and quality of life (highlighting the lack of standardization in outcome measurement). Among them, the ones that were most frequently repeated throughout the studies were: Intrinsic Motivation Inventory (IMI), Simulator Sickness Questionnaire (SSQ), Stroke Impact Scale (SIS), or the Fugl-Meyer Assessment (FMA) for upper/lower limbs. Table 1 shows the main measures in greater detail.

**Table 1:** Comparative summary of gamified stroke neurorehabilitation studies (2019–2023) by technology category

Aspect	Robotic/motorised	Non-motorised	VR/IVR/AR	NMES + games
Number of studies	50	38	68	13
Total number of patients	2.701	988	2.306	409
Typical age	Mostly 50–70 years; somecohorts close to 80	Mostly 50–70 years; some mid-40s and *>*70	Mainly early 50 s to late 60 s; some younger/older cohorts	Mostly early 60 s (approx. 55–70 years)
Stroke phase (Ischemic/Hemorrhagic/Undefined)	1.742/586/37	534/158/296	1.462/203/641	207/69/133
Game environments and interaction	Robots/exoskeletons with SG/SVG; frequent integration with VR/IVR and exergames; some AR-based scenarios; upper limb and gait/balance training	Custom SG/SVG, tangible interfaces, commercial video games and exergames; upper limb/hand, gait and balance, cognition, language and dual tasks	Non-immersive VR, HMD-based IVR, AR overlays and commercial VR exergames; upper limb, gait/balance, cognition and ADL training	FES and tDCS combined with SG/SVG, VR/IVR and sometimes exergames; mainly UL, with some balance/gait protocols
Main clinical outcome domains	Upper-limb motor (FMA-UL, ARAT, WMFT, BBT),disability/ADL (FIM, BI), gait/balance (TUG, 6-MWT, BBS, MBEST),some cognition (e.g. MoCA, FAB), plus kinematics and physiological data	Upper-limb motor/dexterity (FMA-UL, BBT,ARAT,MAL), gait/balance (TUG,6-MWT, BBS,F8W, 10-MWT),ADL/independence, cognitive and neuropsychological tests, language/communication in some studies; frequent game-performance metrics	Upper-limb motor (FMA-UL,ARAT,MAL,WMFT, AoU/QoM),ADL/independence(FIM, BI, IADL,DASH), gait/balance (BBS,6-MWT,TUG),wide range of cognitive/neuropsychological tests; some physiological/neuroimaging markers	Upper-limb motor (FMA-UL, ARAT, AMAT,MI, MAL, handgrip), ADL/independence (BI, FIM-related), and, in some trials, gait/balance (BBS, TUG, BESTest,FGA, 6-MWT); occasional neurophysiological and cognitive measures
Usability, motivation and QoL measures	SIS, SS-QOL, EQ-5D, SF-36/SF−12; SUS, QUEST, UEQ, NASA-TLX, USER-P; IMI, BDI, HADS, PPS;additional adhoc usability, feasibility and enjoyment questionnaires and interviews	SUS, USEQ, VUE-Q, GEQ; SF−36/SF−12, SIS, WHOQOL, SAQOL-39g, EQ-5D; IMI, VAS-motivation/satisfaction, BREQ, RCI; WEMWBS,PHQ, GAD-7; frequent feasibility, frustration and satisfaction questionnaires and qualitative feedback	SUS, USEQ, SSQ, VEQ, TAM, NASA-TLX, IPQ, PGTQ, meCUE; EQ-5D, SF−36/SF−12, Neuro-QoL,, WHOQO, WHODAS 2.0;, PAES, GEQ, TSRQ, VAS-basedscales; BDI, HADS, GDS-30, DASS-21; manyfeasibility, acceptability and satisfaction tools, plus interviews	SUS, TAM and related usability/acceptance scales; IMI, NASA-TLX, PAES,diaries and qualitative feedback; SIS and SS-QOL for stroke-specific QoL; short feasibility and satisfaction questionnaires and narrative reports
Therapy dosage	Typically 2–12 weeks;2–5 sessions/week; 30–60 min/session; plus single-session feasibility or user-experience trials	Single-session feasibility up to 2–8 week programmes; usually 2–5sessions/week, 30–60min/session; some studies defined by a fixed number of sessions (e.g. 10–24+)	From single-session exposures to longer programmes with 10–50 sessions;typically 3–6 weeks,3–5 sessions/week, 20–60 min/session; several extended outpatient or home-based uses	From 1–2 session feasibility studies to 9–12 week programmes; usually 2–5 days/week, 20–60 min/session; some protocols specify a total number of sessions (e.g. *≥*27)

### Clinical and usability outcomes

The 169 studies reported a wide range of clinical and patient-reported outcomes. On the clinical side, the most frequent domains were upper-limb motor function, balance, gait, activities of daily living, and cognition. Typical outcome measures included standard motor scales, functional independence measures and balance or gait scales, among others.

In addition, a large number of studies assessed patients’ experience with the gamified intervention through usability, satisfaction, motivation and quality-of-life questionnaires. Overall, gamified devices were associated with comparable or superior clinical outcomes to conventional therapy in most studies, together with high levels of satisfaction, perceived usefulness and engagement with treatment. However, the diversity of tests (88 different scales. Only 21 of them were used in three or more studies) and the frequent absence of complete demographic or usability data still limit direct cross-study comparisons. However, the review [16] provides an overview of various gamified systems, offering insight into the current state of gamified rehabilitation for stroke patients and highlighting different domains in a comprehensive manner.

These observations are important for the present work because they highlight both the clinical potential of gamified devices and the current limitations in how outcomes and usability are reported, which directly affect the design recommendations derived from these systems.

Table 1 summarizes the main characteristics of the 169 studies included in [16]. More detailed information on these studies can be found in the cited article.

### Relevance for the present work

The present study complements the PRISMA-guided sys- tematic review presented in [16] by conducting a new, technical and design-oriented analysis of a selected subset of gamified systems. From the 169 empirical studies includedin [16], the focus here is on the self-developed, non-commercial devices that provide sufficient information about the gamified interaction, the sensing and actuation technologies, and the implementation of game elements.

For these prototypes, a new coding scheme is applied. Each device is annotated with labels describing: (i) the game environment (e.g. VR, IVR, exergames), (ii) the number and type of implemented games, (iii) the technologies that compose the device (robotics, exoskeletons, sensors, tangibles, HMD, microcontroller-based platforms, etc.), (iv) the game development software, (v) the targeted patient domains and implementation setting (clinic, home, telerehabilitation, or hybrid), and (vi) the gamification components.

According to the Mechanics-Dynamics-Aesthetics (MDA) framework, the gamification components are explicitly distinguished by game mechanics, dynamics, and aesthetics. A detailed description of each game element is provided in **Online Resource 1** (Supplementary Material 1). These descriptions of game components, based on the literature, have helped to codify the various elements analyzed in each gamified system reviewed. As a result, a fully detailed table of all these labels applied to the reviewed works can be found in **Online Resources 2** (Supplementary Material 2).

Based on this coding, the present article offers:

- A detailed characterization of design choices in recent self-developed gamified systems.
- A comparative analysis that highlights dominant and under-represented configurations.
- A synthesis of cross-cutting hardware and software design criteria for future devices.

While [16] mainly examined how gamified therapy has been evaluated in terms of functional, cognitive, emotional and social outcomes, the focus here is on the underlying design of the devices themselves. In this way, the present analysis complements the clinically oriented contribution of [16] by providing an extensive catalog of design labels within a hardware–software design framework. These three contributions (new coding scheme, comparative analysis, and design synthesis) were not part of the original systematic review and constitute the independent added value of the present work.

## Key gamification parameters

**Fig. 3:**
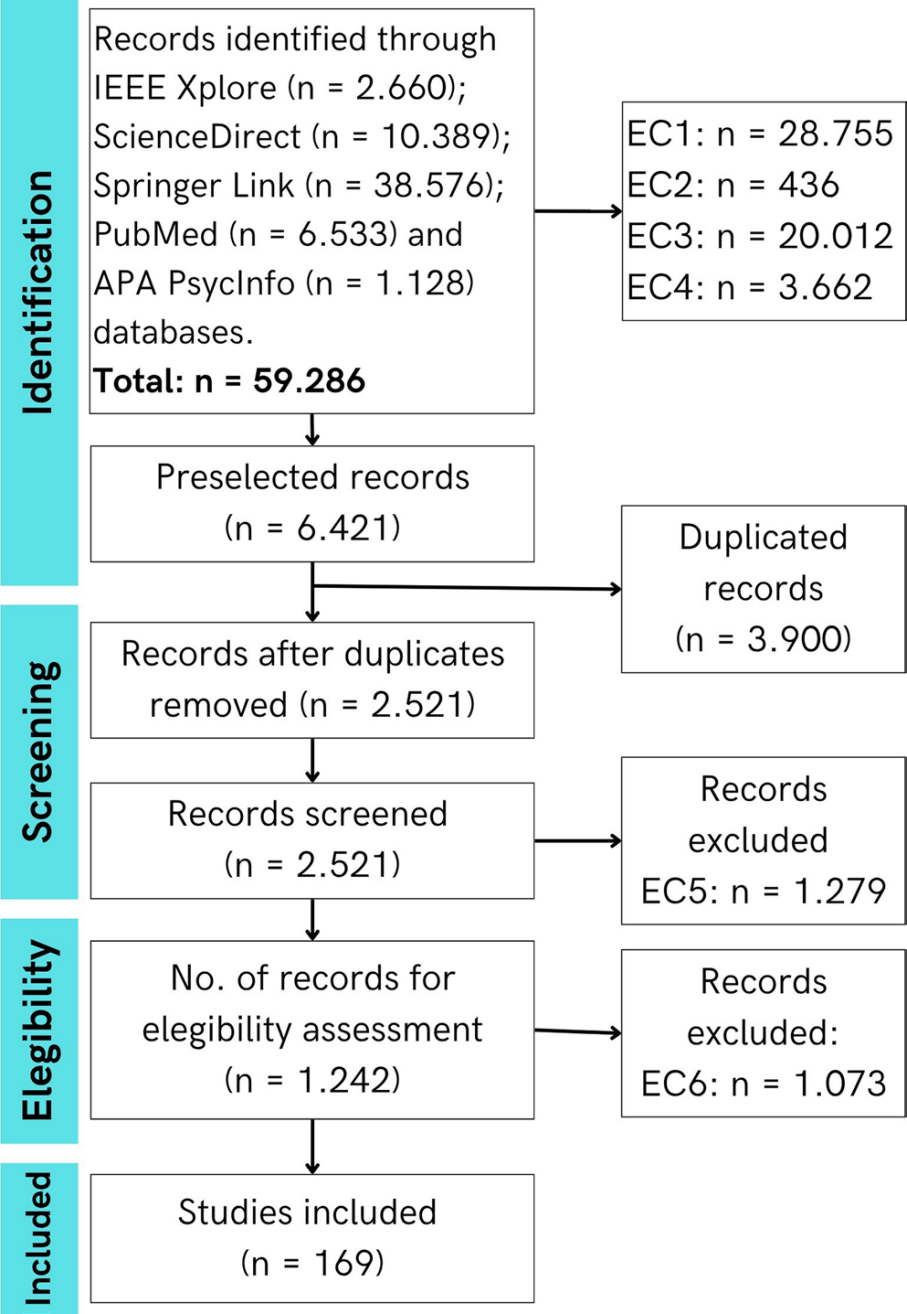
Characteristics and domains of gamified therapy

This section will analyze the key parameters of gamification, synthesized to facilitate a better understanding of the proposed design criteria and a more effective integration of the elements to be presented here in relation. After discussing the essence of gamification in depth, the most frequent theoretical frameworks in which gamification programs are usually situated will be defined. Figure 3 presents a detailed outline of the key aspects that will be described in the following sections.

The figure shows the two broad divisions that can be made of gamified therapy. The first is the patient domain, which is the main focus of rehabilitation. The second is the gamified therapy setting, which in turn is divided according to the interface used (technologies, hardware), the software used (the environment in which the game is developed along with its intrinsic components) and the physical location in which the therapy is implemented.

### Gamification essentials

By incorporating gaming elements into non-gaming environments and contexts, the objective is to achieve an equilibrium between the challenges encountered (in the rehabilitation process, in this case) and the capabilities of the users (patients). Patients typically focus on overcoming their limitations through their engagement in the dynamics of the games. In gamified rehabilitation therapy, one of the critical aspects is the appropriate repetition of the requisite therapeutic exercises [14, 35, 42]. A comprehensive understanding of the principles of gamification facilitates the development of suitable designs to enable this fundamental basis of gamified rehabilitation, while simultaneously promoting increased motivation and engagement [43].

Consequently, the integration of gamification into rehabilitation programs encompasses more than merely the physical improvement of the patient. This limitation was already mentioned previously, in the proposal. As emphasized in [44], gamification has the potential to impact four patient dimensions: (s) affective, (b) cognitive, (c) physical/motor, and (d) social. Traditionally (as seen before), the literature has focused predominantly on the physical and cognitive dimensions of gamified rehabilitation, neglecting other significant aspects. This imbalance is highlighted in [16], where an effort is made to analyze how gamification also exerts a substantial impact on the affective and social dimensions.

Given the aforementioned considerations, and upon examination of the relevant literature, it is appropriate to consider the integration of **cognitive** and **physical/motor **levels into a single domain, as these approaches are frequently complementary and encompass exercises for enhancing Activities of Daily Living (ADL) [6, 14, 16, 45]. This consolidated concept may be termed the **functional** level, as it incorporates both cognitive and motor aspects while emphasizing the restoration of skills crucial for autonomy in the patient’s daily activities. 

Consequently, the resulting levels would comprise: (1) **affective**, (2) **functional** (integrating cognitive, motor, and ADL), and (3) **social**. This revised categorization not only more accurately reflects the nature of gamified interventions but also facilitates the development of more comprehensive and effective rehabilitation programs. 

These three pillars (affective, functional, and social) are fundamental to the design and evaluation of gamified therapies. Such therapies have the potential to enhance patients’ quality of life through these pillars: neglecting one may compromise the entire intervention. While it is feasible to develop SG focused on cognitive or motor rehabilitation, it is imperative to acknowledge the significance of gamification in improving the affective and social dimensions as well.

### Gamified therapy setup

While the previous emphasis was on the patient level directly affected by gamification, gamified therapy can also be analyzed by focusing on three key aspects that facilitate understanding of its implementation and impact on patients (Fig. 3): (1) technology, (2) game environment, and (3) therapy implementation. These three aspects are fundamental for comprehending the application of gamification in neurorehabilitation. A thorough understanding of the interaction among these elements will significantly influence the efficacy of gamified design and interventions. Some of the distinctions presented below were briefly addressed in [16], which were also discussed in somewhat greater detail in [14].

#### Interface (Technology)

Encompasses the devices and physical components that facilitate the gamification of therapy. This includes both motorized technologies (robots, exoskeletons, motorized treadmills, etc.) and non-motorized technologies (tablets, wearable devices, Kinect (Microsoft Corporation), tangible objects, gloves, sensors, etc.), as well as advanced technologies such as Head-Mounted Display (HMD) devices, Brain–Computer Interface (BCI), and NMES. This distinction has already been discussed in [26]. Interface (technology) can be considered the “hardware” of gamified therapy, as it comprises the physical devices that enable interaction between the patient and the gamified environment. The design criteria are developed in more detail in Sect. 6.

#### Software

This constitutes the medium in which gamified experiences are developed. It encompasses the **Games Environments**: immersive technologies such as VR, which is a comprehensive term further categorized into Augmented Reality (AR) and Inmersive Virtual Reality (IVR) (based on the level of immersion); tangible Serious Games (SG) (incorporating interactive or tangible physical elements), Serious Virtual Games (SVG) (which are SG developed in a virtual environment), exergames, and commercial video games. These environments can also be analyzed within the MDA framework [46–49]. These three components form the traditional foundation of the Game Environment and are crucial for appropriate design. This collective framework constitutes what may be termed the “software” of gamified therapy, as it provides the environment in which the patient interacts with the treatment.

More specifically, the framework MDA is defined by:

- **Mechanics**: These are the rules and systems that guide the player’s actions [46, 48–50]. They are the mechanisms by which the player (in this case, the patient) interacts with the system and responds to the challenges presented. They ensure that therapeutic tasks are not only completed, but done in a way that promotes motor, cognitive, neuro-plastic learning or skill enhancement. Examples might include: timings, goals and tasks, etc.
- **Dynamics**: These refer to the driving forces that drive the interaction of the player or patient over time [3, 46, 48–50]. They are the general behaviors that are generated as a result of the rules and systems of the game. These dynamics are key to encouraging participation and keeping the patient motivated and engaged, as they also set the tone and pace of the patient’s experience. Some examples are: narrative, game modes (multiplayer, single player…), progression, etc.
- **Aesthetics**: is the component responsible for eliciting and modulating the emotional and intellectual response of the player through sensory feedback (graphics, sound, haptic effects) and the deliberate incorporation of stimuli [46–49]. In a rehabilitation context, this dimension is designed to keep the patient engaged and motivated, promoting feelings of excitement, achievement and well-being, and fostering self-efficacy in the self-care of their disease.

Some of the most common game elements found in the literature that define the most basic software design of the Serious Games (SG) are: difficulty, voting, goals, tasks, challenges, feedback, unlocks, rewards, speed, time, lifes, rules, help, adaptability, progress, simplicity, game mode, chat rooms, story, narrative, leaderboards, score, points, levels, awards, badges, Non-Playable Character (NPC), avatars, customization.

All of these elements are key to properly understanding how SG are composed. A more comprehensive review is provided in **Online Resource 1** (Supplementary Material 1). A good understanding of their nature and their potential integration is key when considering the software design of a SG in any of its game environments.

#### Therapy implementation

The location and method of delivering gamified therapy are crucial considerations. Several options exist, including rehabilitation conducted at home without direct clinical oversight, telerehabilitation that combines home-based treatment with remote clinician interaction, and therapy administered in clinical or hospital settings under the immediate supervision of healthcare experts. The choice of setting significantly impacts the design and efficacy of gamified therapeutic interventions. Each approach presents unique considerations that influence how these game-based treatments are developed and implemented to achieve optimal results.

Integrating these three aspects (technology, game envi- ronment, and therapy implementation) along with the dis- tinction between “hardware” and “software” provides a more structured framework for analyzing and designing gamified therapies. Synthesizing: hardware refers to the tangible devices that interact with the patient, while software represents the environment in which the gaming experience occurs. These two components are inextricably linked and must be adapted to the environment in which they are utilized: at home, through telerehabilitation, or in a hospital or clinical setting. This framework offers a more structured and coherent perspective on how gamified technologies can influence the design and efficacy of rehabilitation.

Understanding these three aspects facilitates the transition from theoretical concepts to practical applications. Prior to designing a device, it is essential to comprehend all these facets to understand the intrinsic characteristics of the therapy. This approach enables proper categorization of hardware, with various options depending on the preferences, capabilities and requirements of each rehabilitation team. Furthermore, knowledge of the elements of gamified therapy software allows for the evaluation of patients’ intrinsic preferences in relation to the game. These considerations will be examined in greater detail in subsequent sections.

## Current trends analysis

This section will present the new trends found in the gamified design of post-stroke therapy between 2019 and 2023. These works have been studied in [16] from a holistic perspective of patients, taking into account their three domains: functional, social and affective. For this reason, they are considered suitable to be the subject of a study from a purely design perspective.

Having a good understanding of how the most novel designs have been approached (a total of *n* = 68), putting them under the focus of all the features and essences of gamification seen in Sect. 4, will be key to see the latest trends in gamified design. Finding possible shortcomings, insufficient approaches or lack of adaptation will be key to see the need and elaboration of the proposed scales in the following chapters.

To ensure a correct analysis, if any of the novel design articles reviewed are based on previous studies where they describe in more detail the gamified technologies employed, these previous works have been consulted to acquire all the key features. It should be noted that the features and ele- ments analyzed were those that were made explicit in some way in the reviewed studies. It is important to note that only studies presenting own designs (not those based on commercial devices) were considered in order to identify emerging trends in new prototypes; moreover, complete and detailed information on commercial devices is not fully available.

Table 1, founded in **Online Resources 2** (*Supplemen- tary Material 2*), shows all the reviewed studies in [16], analyzed under the essential characteristics of gamification already described. The statistics found in the new gamified trends in stroke neurorehabilitation are described below. Although the focus is on stroke, it should be noted that some of the works reviewed in [16] included in the experimental groups patients with different neurological pathologies, such as multiple sclerosis. Therefore, as previously mentioned, all the analyses described in this proposed work can be extrapolated to neurorehabilitation therapies and the gamified technologies used in them.

### Patients domains

**Fig. 4:**
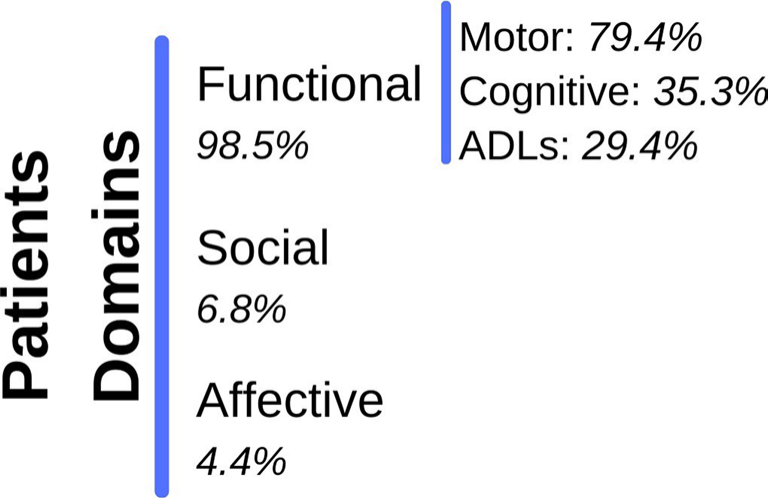
Total percentage of works and domains covered in the new gamified devices (*n* = 68)

There are three domains of the patient that gamified neurorehabilitation therapies target: Social, Affective, and Functional (which includes: Motor, Cognitive, and ADL). Gamified therapies should be designed to improve these three patient domains, all of which are essential. Figure 4 shows a summary of the main domains found in Table 1 (**Online Resource 2**).

The analysis shows that the vast majority of gamified technologies in neurorehabilitation are focused on functional rehabilitation (98.5%): 79.4% motor rehabilitation, 35.3% cognitive rehabilitation, and 29.4% ADL rehabilitation. However, although the social (6.8%) and affective (4.4%) domains are incorporated into some gamified solutions, they do not constitute the primary focus of most current devices.

While certain devices integrate multiple domains, functional rehabilitation remains the predominant priority, presenting opportunities for future research to more effectively incorporate social and emotional aspects into the design of these technologies. This situation presents a critical line for the future of gamification in neurorehabilitation: a more comprehensive approach that not only enhances patients’ physical functionality but also improves their motivation and social and emotional well-being, thereby contributing to more holistic and sustainable rehabilitation, as observed in [16].

### Setup of gamified devices used in neurorehabilitation therapies

This section discusses how the configurations of the new gamified devices were carried out, analyzing the technologies used, the most commonly employed development software, the most recurrent games environments, and gamification components.

#### Technologies (Interface)

**Fig. 5:**
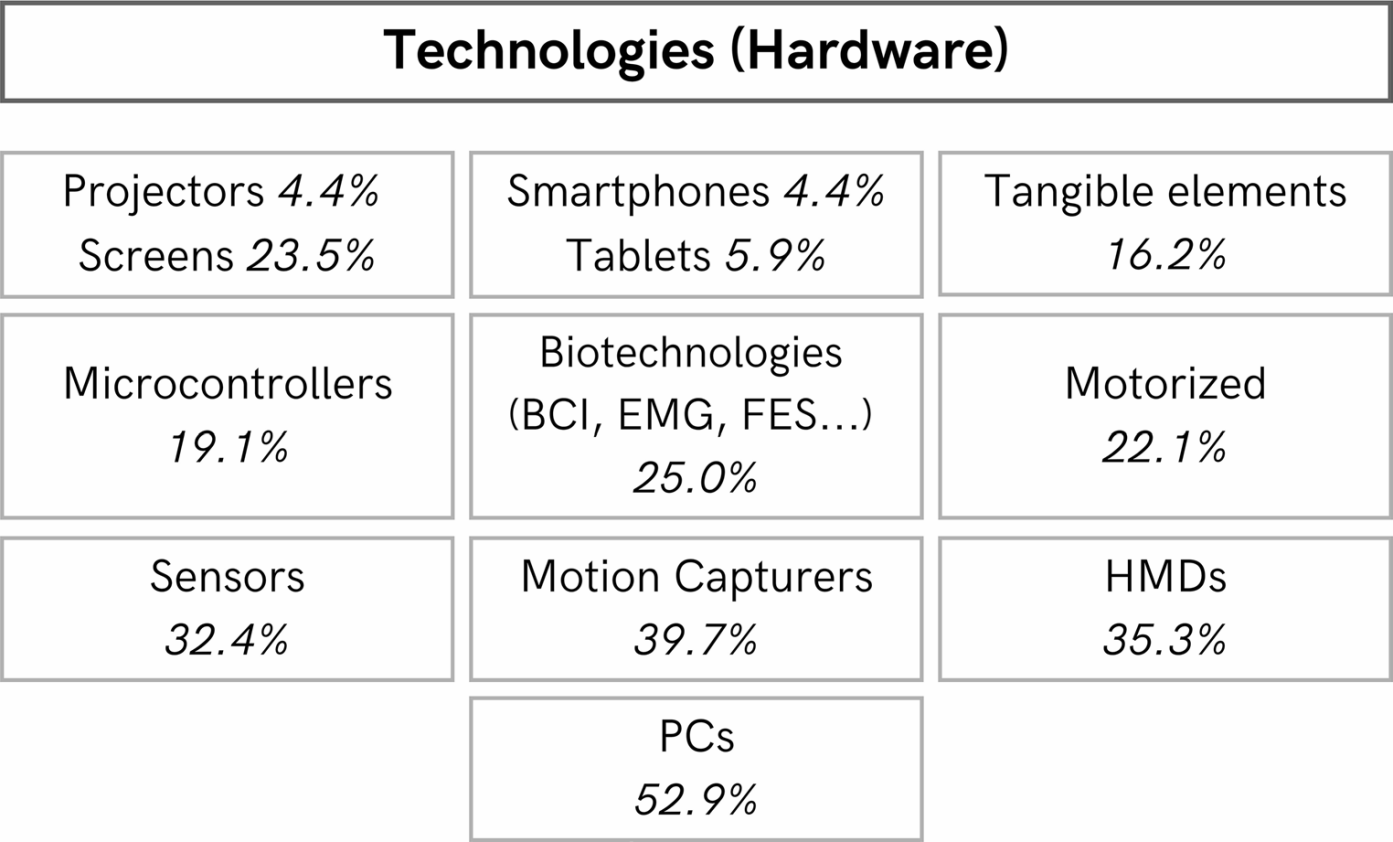
Percentage of the main technologies found in the re- viewed studies (*n* = 68)

Starting with the technologies used (the interface of the gamified therapy), a wide variety is presented in Table 1 (**Online Resource 2**). Figure 5 shows a summary of the main categories found. It is important to note that many of the gamified devices combined various technologies.

Among them are motorized technologies, used in 22.1% of the reviewed studies. From the total of them: treadmills (33.3%), gloves (20.0%), motors/actuators (20.0%), exoskeletons/orthoses (13.3%), and robots (13.3%). Additionally, one study employed a stationary bike.

Among more accessible everyday technologies, tablets were used in 5.9% of the studies, smartphones in 4.4%, and PCs in 52.9%. Furthermore, 23.5% used screens (with 3 of them being touch screens) and 4.4% used projectors. Finally, 5.9% involved solutions that used desks on which the patient interacted with the device. These data suggest that common device solutions remain an important option for ensuring accessibility in game-based rehabilitation.

On the other hand, 7.4% of the studies incorporated BCI technologies, while EMG was also used in five studies. Then, 2.9% employed EEG, and 1.5% used ECG. Lastly, 5.9% utilized FES technologies. While the use of these more advanced technologies is seen as limited, it is growing. This may open up opportunities for personalization and more accurate patient monitoring.

A total of 16.2% used tangible elements as the basis for the patient’s interaction with the game, while another 32.4% employed sensors. Among these sensors, 45.5% were IMUs, 22.7% were FSR, 9.1% were Load Cells and RFID, and 4.5% were bends, velostat, and manometer. Additionally, in 2 studies, the Xbox 360 controller was used, while in another the Oculus Controller (Meta Platforms, Inc.) and a wearable controller were employed. This shows that the trend towards tangible interactivity continues to be a fundamental element in today’s gamified devices.

Furthermore, 7.4% used cameras, and 13.2% used Kinects. Among other motion captures, 13.2% used the Leap Motion Controller (Ultraleap Ltd), and 1.5% employed the Vicon Nexus (Vicon Motion Systems Ltd) and the Qualisys AB 3D (Qualisys AB). Additionally, two other studies used the HTC Tracker (HTC Corporation). The use of these technologies demonstrates the trend toward accurate tracking of each patient’s movements.

Microcontroller-based solutions were used in 19.1% of the studies. From the total microcontrollers used, 53.8% studies used Arduino technology (Arduino LLC), with 71.4% using Arduinos MEGA, 14.3% RFDuino, and Arduino NANO. Then, a 7.7% of the studies employed: ESP32 (Espressif Systems), or Atmel 32-bit ARM Cortex M3 (Microchip Technology Inc.) or OpenBCI board (OpenBCI) or Teensy 3.2 (PJRC) or Raspberry Pi Zero (Raspberry Pi Foundation). One was unspecified. The widespread use of Arduinos reflects their accessibility and versatility in the design of gamified systems.

In 35.3% of the studies, HMD technologies were used, among which: 50.0% were HMD Oculus (Meta Platforms, Inc.), 29.2% HMD HTC VIVE (HTC Corporation), 4.2% were HMD Valve (Valve Corporation) or HMD Meta Quest (Meta Platforms, Inc.) or HoloLens (Microsoft Corporation). Two other HMDs were not specified in terms of the brand used.

Finally, in 7.4% of the studies, the authors included safety elements as an essential part of the hardware.

All of this data suggests that while advanced technologies such as BCI and HMD are gaining ground, accessible PC and microcontroller-based solutions such as Arduino re- main critical. In addition, accurate motion capture and tangible interactivity are essential to improve the effectiveness of gamified rehabilitation systems. Immersive technologies and safety considerations are consolidating as key elements for future developments.

#### Software

This section will first address various aspects related to development environments, before moving on to the software components in gamified therapy: the Game Environments and their corresponding Gamification Components (mechanics, dynamics, and game elements) that are most frequently used.

- **Development software**

Starting with the development environments used to create the video games, a total of 33 different environments were identified.

Beginning with Unity (Unity Technologies), it was found to be used in 51.5% of cases, employed for creating both 2D and 3D video games. Following that, other development environments such as Visual C# (Microsoft) (7.5%) and Unreal Engine (Epic Games) (4.4%) were also used. Finally, the following development environments were used in 1.5% of the studies: MATLAB (MathWorks), Blender (Blender Foundation), Maya (Autodesk), and Android Studio (Google). In 22.1% of the studies, the development environment was not specified, while in 1.5% of the studies, it was not applicable since the SG were purely tangible, without digital environments.

Lastly, in 29.4% of the studies, the authors explicitly mentioned the development of a GUI to facilitate user interaction with the developed games.

b. **Games environments**

**Fig. 6:**
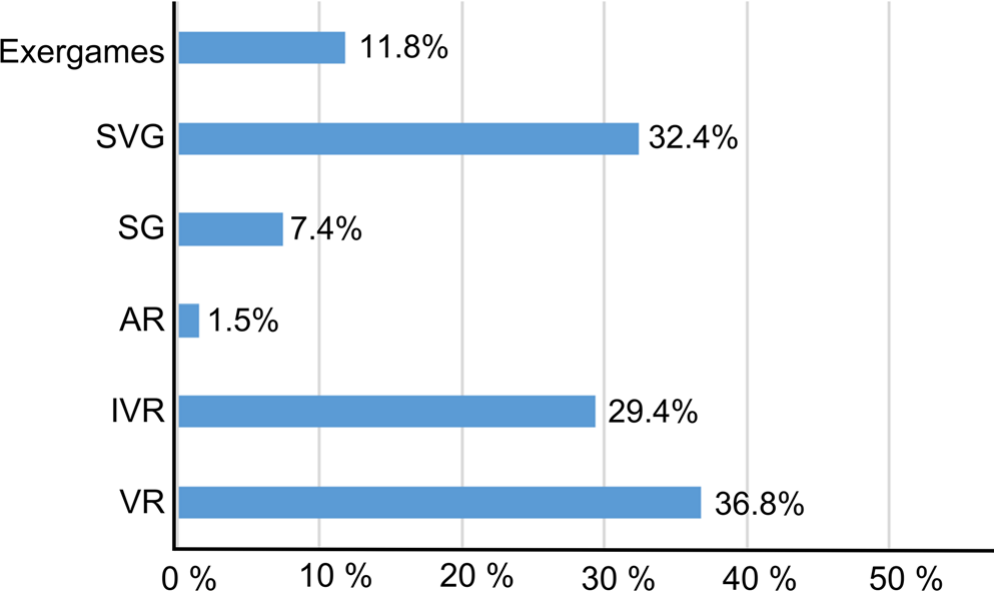
Most common g*ame environments* elements (*n* = 68)

This section will discuss both the average number of games and virtual environments found in each novel gamified de- vice as well as the most commonly used Games Environments. Figure 6 provides a summary of these.

First, in 66.2% of the devices games were implemented, with an average of 3.33 games per device and a standard deviation of 2.64. This mean value should be considered indicative for future designs, as a system with fewer than three games implemented could be deemed insufficiently comprehensive in terms of user experience and motivation, as it would not provide adequate variety or challenge. Therefore, it is advisable for future designs to incorporate a minimum of three games per device to ensure that patients have a more diverse and enriched experience, which may contribute to enhanced engagement and therapeutic efficacy. However, the standard deviation suggests that some devices may benefit from the inclusion of additional games, particularly if further customization or increased challenge is desired for users with varying needs and preferences. This aspect is examined in greater detail in the subsequent sections. The maximum number of games incorporated into a single device was 10.

On the other hand, virtual environments (such as kitchens, supermarkets, stores, etc.) were implemented in 33.8% of the devices, with an average of 1.7 environments per device and a standard deviation of 1.67. For future designs, it would be advisable to incorporate a minimum of two virtual environments in gamified systems, which could enhance the diversity of experiences and render the system more appealing and adaptable to various therapeutic and daily life scenarios. The incorporation of additional environments could be particularly advantageous for users with specific requirements, such as cognitive, ADL or motor rehabilitation, offering more complex and contextualized scenarios that promote motivation, immersion, and functional learning. The maximum number of virtual environments was 7.

Moreover, delving deeper into the Game Environments, the following exclusive implementations were found: 36.8% were VR, 32.4% were SVG (two of which were tangible), followed by 29.4% IVR. Then, 7.4% incorporated tangible SG, and only 1.5% included AR. Lastly, 11.8% included exergames. Of all these solutions, 12 combined several Game Environments within a single device: VR + exergames: 33.3%; IVR + exergames: 25.0%; VR + IVR: 16.7%; and in 8.3% SVG + VR or SVG + SG or SVG + exergames were found. These combinations may reflect a new trend toward the design of more complex and adaptable solutions, underscoring the flexibility and diversification in the development of these technologies to meet different needs.

c. **Gamification components**

This section will detail the Gamification Components that made up the most common and widely used Game Environments.

Starting with Mechanics, Table 2a shows the frequency of the most commonly used ones (total *n* = 68). There is a prevalence of Feedback (98.5%), which was visual in 94.0% of the cases, 43.3% as auditory, 13.4% as haptic, 13.4% as postgame feedback, 5.9% as FES, and 1.5% as neuro feedback. An additional element subjected to analysis was time, which was utilized in 45.6% of the cases: in 64.5% studies this mechanic was implemented as a countdown while in 16.1% it was used to measure time spent. In 22.6% studies, the type of Time mechanic was not specified. On the other hand, the Help mechanic was mainly implemented in the form of player instructions. The Rules are elementary (100% of the cases) to adequately define a game.

**Table 2:** Frequency of g*ame components* reviewed (mechanics, dynamics, and game elements). Total (*n* = 68)

(a) Most used Mechanics	
Mechanics	Frequency (%)
Difficulty	32.3
Voting	0.0
Goals/Tasks/Challenges	82.3
Feedback	98.5
Unlocks	2.9
Rewards	63.2
Speed	17.6
Time	45.6
Lifes	7.4
Rules	100
Help	47.1
(b) Most used Dynamics	
Dynamics	**Frequency(%)**
Adaptability	29.4
Progress	32.4
Simplicity	27.9
Game Mode	100
Chat Rooms	7.4
Story/Narrative	10.3
(c) Most used Aesthetics Elements	
AestheticElement	**Frequency(%)**
Leaderboards	8.8
Score/Points	60.3
Levels	30.9
Awards	2.9
Badges	0.0
NPC	23.5
Avatars	47.5
Customization	8.8

Moving on to Dynamics, Table 2b shows the most commonly used ones. As expected, Game Mode is present in each and every new design, as it determines how the patient engages in the game. The most common modes were singleplayer (94.1%), multiplayer (4.4%), and both modes were found in one study. Additionally, competitive dynamics were introduced in four studies, collaborative dynamics in three, and in two studies, the Game Mode translated into Player versus Environment (PvE).

Finally, the most commonly used Game Elements are listed in Table 2. The Rewards mechanic materialized in 60.3% as Score/Points, and in 2.9% as Awards. The Aesthetic element was also found in 94.1% of the studies. The main aesthetic considerations were as follows: 85.3% implemented 3D Aesthetics, while 27.9% integrated 2D Aesthetics. Then, 25.0% were realistic (three of them hyper-realistic), while 19.1% were simple. In 5.9%, the games were designed in a classic style. Additionally, 42.7% were in first person perspective, while 10.3% were in third-person perspective. Regarding NPCs, which appeared in 23.5% of the studies, they were introduced as hostile in four studies, as allies in two, and as neutral in nine.

It should be noted that some mechanics and dynamics, such as Goals/Tasks/Challenges, and Progress, are often considered obvious and therefore not explicitly mentioned in the descriptions of gamified devices. For this reason, more of these have not been included, however obvious they may be, as they could not be known during the review.

#### Therapy implementation

Lastly, the main environments where gamified therapy has been implemented will be detailed. First, 82.3% of the studies implemented the devices in a clinical setting. On the other hand, 10.3% were in-home implementations without teleassistance, while 8.8% were telerehabilitation. Finally, it is worth noting that eight of the devices had implementations that could be combined both in the clinic and at home. Specifically, five studies combined clinical implementation with telerehabilitation, while three studies combined clinical and in-home implementations.

## Design considerations

Having reviewed the current trends in a comprehensive manner, this section will outline some of the most commonly encountered hardware and software design considerations for gamified systems. From general criteria (which should be applied or considered for all systems) to more specific ones, such as robotic technologies, sensors, VR, and software.

### Generic hardware design criteria

The hardware utilized in gamified therapy (motorized or non-motorized) plays a fundamental role as it serves as the medium through which the patient interacts with the rehabilitation environment, as mentioned above. It also largely determines which movements can be trained, at what intensity, and in which settings (hospital, outpatient clinic, or home). The selection and design of this hardware must be carefully considered, taking into account both the specific needs of the patient and the therapeutic objectives. The hardware must not only provide precision in measurement and response [26], and facilitate rigorous exercise, but also be accessible and suitable for the clinical and rehabilitation environment.

As previously discussed, and as detailed in [14], each hardware will have its characteristics, advantages and disadvantages. In the study here presented, this diversity translates into a wide range of configurations, from simple PC-based systems to complex robotic platforms, which reinforces the need for context-dependent hardware selection rather than a ‘one-size-fits-all’ approach. Depending on the context of each clinic/hospital or home, the patients and the preferences of the clinicians, an appropriate selection of hardware should be made. It has been widely demonstrated that either robotic therapy, tangible SG/SVG or VR therapy is effective for both subacute (less than six months since episode) and chronic (more than six months since episode) stroke patients [14, 16, 45, 51].

As have been seen, the hardware used are usually devices such as HMD, assistive/passive/resistive robots, treadmills, exoskeletons, tactile elements, sensors (the most commonly utilized are accelerometers and gyroscopes), Kinects, wearables, haptic gloves, among others.

**Fig. 7:**
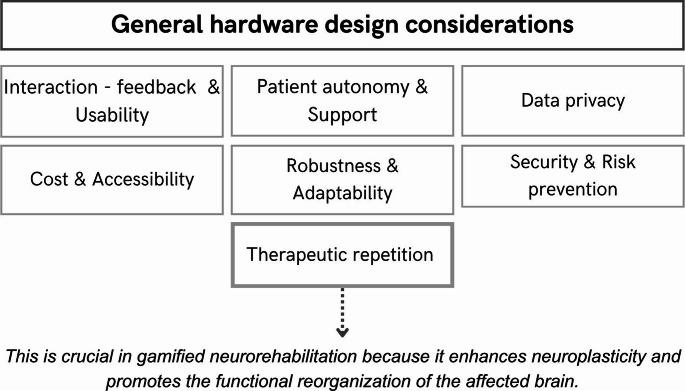
Key considerations for designing the interfaces of gamified systems

In addition to the research presented in [14], which describes the benefits and limitations of different types of devices, the following general considerations should be taken into account when designing the hardware of gamified systems for neurorehabilitation. An outline of the considerations described here can be seen in Fig.7.

1. The user interface must be intuitive and with a learning curve that is not too complex [27, 31, 52–60]. In this regard, it should also be considered whether initial training is required for either the rehabilitation team or the patient and adapted to their needs [40, 61–64].
2. In addition to the above, another consideration is to take into account the potential autonomy of the patient to be able to perform the movements with the device in.

an autonomous manner. Also, everything related to the installation of the systems: electrical connection, physical space, among others. Finally, when considering the autonomy of the patient, consider an adequate technical support integrated in the system [27, 28, 34, 58, 62, 65]. In practice, this means clarifying at the design stage whether the device is intended for supervised use in a clinical setting, partially autonomous use at home, or whether it is configurable for both, as this will determine both the complexity of the hardware and the security requirements.

3. The equipment must ensure that it is safe for the pa- tient to perform therapeutic movements. It must prevent incorrect movements, fatigue, misuse or possible falls [28, 31, 41, 52, 58, 60, 62, 66–68].
4. Along with Criterion 3, the gamified hardware must be prepared for intensive repetition of the therapeutic exercises [28, 31, 35, 42, 53, 69, 70]. This is especially relevant in rehabilitation after a stroke, where high-intensity, high-repetition training is associated with better functional outcomes. Therefore, mechanical robustness or the ease of resetting the device (e.g., game variables, specific configurations) between trials becomes critical.
5. Usability: Devices should be easy to use for both the patient and the therapist [31, 71]. They should also be comfortable to use [26, 28, 41], robust [28, 41], and made of appropriate materials that ensure both lightness and ergonomics [40, 41, 70]. In addition, consideration should be given to the patient’s limitations for gripping, as mentioned in [6].
6. Cost and Accessibility: Although high-tech devices offer many benefits, it is also important to consider their cost and accessibility [26, 28, 31, 40, 52, 61]. Gamified rehabilitation must be accessible to a wide range of patients, so evaluate options that are efficient without compromising cost. In addition to patients, it must also be cost-effective for clinical centers. This review shows that low-cost solutions using commercial hardware and microcontrollers are already being explored. This suggests that economic barriers do not necessarily preclude the development of clinically useful gamified interventions, provided that the aforementioned design criteria are met.

7. Incorporate an objective evaluation system based on the patient’s game performance to complement clinical testing and medical follow-up. This objective assessment should be accompanied by clear and timely feedback for both the patient and the therapist. For the patient, this feedback should include, for example, progress indicators, awareness of results and performance, and error warnings. For the therapist, this feedback should include automatic reports and trends over time. This feedback should guide therapeutic decisions and adjustments during the session. In addition, consider how the system can support and adapt to the patient’s performance to adjust the parameters of the gamified therapy, such as speed, difficulty, or other game dynamics [26, 28, 42, 53, 58, 64, 72].

8. The system must ensure the privacy of the patient’s personal information [28, 54, 61, 66, 72].

In summary, the design of hardware for gamified therapies must balance clinical accuracy, safety and usability with practical considerations such as affordability and ease of installation. For design teams, this summary can be used as a checklist when specifying new devices or selecting among existing options for a particular clinical programme. Only by integrating intuitive interfaces, ergonomic materials, objective evaluation systems, and technical support mechanisms can the technology not only enhance rehabilitation effectiveness but also adapt to the realities of patients, therapists, and healthcare facilities.

#### Motorized, robotics and exoskeletons

Robotic rehabilitation systems are usually high-precision systems [26, 32] that are classified according to their mechanical structure, actuation principle, training mode, and place of use [60, 73].

Structurally, there are end-effector-based systems, which interact with the patient through a single distal fixation point to allow exercises in the Cartesian XYZ space. There are also exoskeleton-based systems, which envelop the whole arm to control its orientation and coordinate complex move- ments. In rehabilitation therapy this is key for training specific, assisted, and repetitive movements [31, 60, 70, 74].

According to their actuation principle, robots can be passive (the robot follows a predefined path without generating its own force), active (the robot itself produces the movement), or interactive (they adjust their assistance in real time to the patient’s response).

Regarding the training mode, they offer passive movement (the robot moves the limb without patient effort), assisted movement (the robot supports the movement according to the patient’s residual capacity), or resisted movement (the robot provides resistance to strengthen the musculature).

Patients who use gamified robotic systems may benefit if these technologies include direct feedback that guides the user’s actions [26, 53, 60]. This benefit can be enhanced by combining the systems with sensors to optimize the rehabilitation process and monitoring [41].

In addition, motorized systems should not be overly complex to use, in order to improve their adaptability to the patient’s abilities [26, 31, 52, 60, 74]. This must be considered with respect to the level of patient autonomy expected, as has been mentioned above, since these systems usually require professional supervision [26].

Furthermore, physical space, device weight, and ergonomics must be considered [31, 52, 74]. Potential misalignments that the system may experience, and ways to resolve them, must also be addressed [41, 52, 74]. Additionally, when designing a robotic system, the appropriate study of the device’s degrees of freedom according to its applications and the target muscle or limb for rehabilitation must be very clear [40, 41, 52, 74]. This may include an explicit justification of which joints are actuated and which are left free, as well as an explanation of how this configuration supports specific functional tasks (e.g., reaching and grasping, gait training).

Finally, although it has already been mentioned, costs are key when designing a robotic system, as its accessibility is usually conditioned by the price of these technologies [26, 32, 40, 52, 60, 70].

**Fig. 8:**
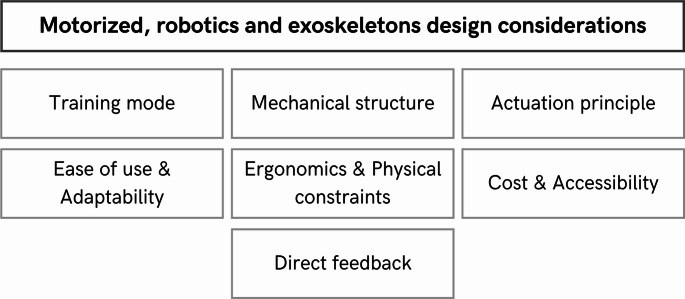
Key considerations for designing motorized solutions

Figure 8 shows a synthesis of everything seen here.

#### Sensors

The sensors most commonly used to monitor a patient’s therapeutic movements are, according to [29], motion sensors (accelerometers, IMU), force sensors (pressure, flexion, deformation), bioelectric sensors (EMG, EEG, Magnetomyography (MMG)), and visual sensors. As noted in Sect. 5.2.1, this technology is widely employed in many current gamified devices. The combined use of heterogeneous sensors (e.g., IMU + EMG) enables a multimodal characterization of movement and opens the door to sensor fusion and deep-learning strategies, enhancing the specificity of therapeutic feedback.

One of the first criteria to consider is undoubtedly sensor placement. This topic is studied in depth by the authors of [29, 75]. In [29], they also evaluate the integration of deep learning and machine learning with sensor readings. Proper placement is crucial for monitoring and data acquisition for subsequent processing [29, 54, 61, 72, 75, 76]. Improper placement can result in misleading information for game processing. Therefore, the sensor position must be validated by physicians as well as technically to ensure that it reflects clinically meaningful movements.

Undoubtedly, the choice of sensors is extensive in the lit­erature thanks to their low cost [57, 72, 76], the portability they offer the patient (non-invasive) [54, 57, 61, 70, 72,  75, 76], their adaptability [75, 76], and their accessibility [57, 61, 72].

In this regard, patient comfort must be taken into account [72], since poor or excessive wiring can be uncomfortable [75]. In addition, they can reduce the patient’s autonomy and mobility, although wireless systems are more comfort­able, but battery and bandwidth must be considered [75].

On the other hand, although [75] emphasizes the accu­racy of sensors when capturing patient signals, several neg­ative considerations must be addressed when using them. First, sensors can suffer from interference issues [75, 76]. In addition, IMUs (the most common sensor type, as already noted) can experience drift problems [75, 76]. It should also be noted that accuracy can be variable for certain data [54, 61, 70]. These issues can be mitigated in the following ways (1) implementing filters (such as the Kalman filter), (2) simple calibrations, and (3) secure sensor mountings. From a game design perspective, reducing noise is essential to avoid situations where patients perceive the system as “unfair” because their on-screen performance does not match their actual effort.

These criteria are key when planning the design and de- velopment of new gamified technologies aimed at optimizing the integration of both the software that will process the data collected by the sensors and its subsequent interaction with therapeutic games.

**Fig. 9:**
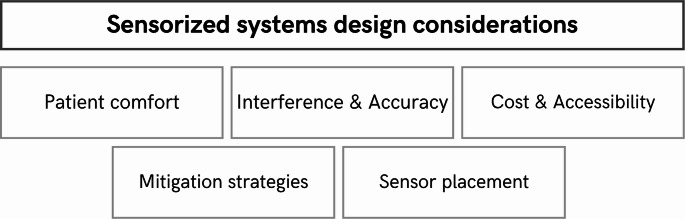
Key considerations for designing sensorized solutions

Figure 9 shows a synthesis of everything seen here.

#### Virtual reality (VR) systems

Virtual reality has increasingly adopted an important role in gamified stroke rehabilitation. Compared with more complex gamified systems, Virtual Reality (VR), Inmersive Virtual Reality (IVR), and Augmented Reality (AR) are more affordable [26, 64, 66]and easier to use [26], which explains their widespread adoption. Commercial solutions based on VR have also been regarded as ideal for telerehabilitation [26, 70]. However, one must also consider the high initial investment required (although these systems can later become cost-effective by reducing medical staff time and costs) [59, 63].

Among other advantages of VR systems is their potential for a simple interface and realistic simulations of activities of daily living. This immersiveness is a key reason for the broad adoption of this technology in studies with neurological patients [57, 62, 67, 69], in addition to its adaptability, personalization, and flexibility [58, 59, 62–64, 67, 69, 77]. They also typically offer multisensory interaction, which not only facilitates direct feedback but also has the potential to induce high motivation (and, consequently, greater adherence to therapy) [58, 59, 63, 64, 67, 68, 70, 77].

While all these considerations are key when deciding whether to invest in this technology or when designing around this game environment, a number of drawbacks associated with the use of VR must be taken into account.

First, in many cases these systems have lower precision than others, as they tend to be more susceptible to environ- mental factors [26, 57–59, 66, 68].

Next, these technologies may be unsafe for frail patients [57, 62, 66], potentially leading to cognitive overload andcybersickness [57–59, 62, 64, 66, 69]. The hyperrealism that many IVR-based games present can also provoke the “uncanny valley” effect [48, 49, 64], in which the simulation is so realistic that it becomes unsettling. It should also be noted that HMD devices can be uncomfortable for some patients [57, 58, 69]. Therefore, detecting vestibular problems (and the possible resulting cognitive overload) and offering alternative, non-immersive modes can be important design strategies when treating people with significant deficits or older adults who have survived a stroke.

Finally, the heterogeneity of VR systems prevents the standardization of dosage and protocols in gamified neurorehabilitation therapies.

All these considerations are necessary for the preliminary study of the needs to be met and the characteristics of the rehabilitation, the patient, and the environment in which it will take place. Understanding the pros and cons allows for the design of increasingly robust, personalized, and effective devices for gamified stroke rehabilitation.

**Fig. 10:**
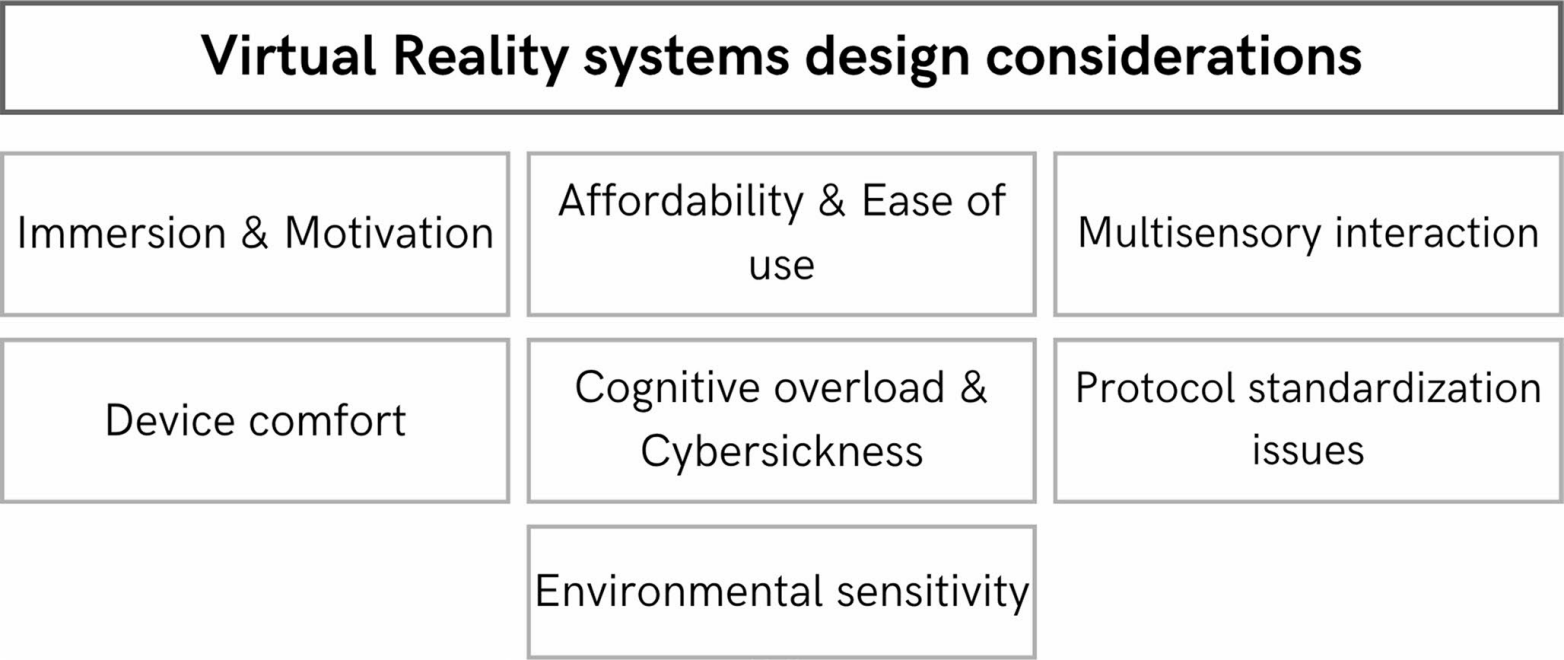
Key considerations for designing Virtual Reality (VR) solutions

Figure 10 shows a synthesis of everything seen here.

#### Exergames

Exergames can benefit from various game environments, such as VR [64, 78]or other interactive games [79], combining physical exercise [36]. They typically foster adherence by including challenges, levels, and rewards characteristic of the gamification methodology [32].

When designing a system based on exergames, it must be noted that they are usually accessible and familiar, as they commonly employ commercial consoles [80]. This makes them especially suitable for community or home-based pro- grammes where equipment and supervision are limited. This makes them more economical than other systems [79] andcan allow more personalized adaptability for the user [80, 81]. However, the commercial hardware itself (as previously noted for VR) may be limited in terms of system accuracy and may require direct supervision at some point [80].

Another consideration is the capacity these systems must have to perform intense, high-intensity therapeutic physical exercises [79]. Potential discomfort for the patient must also be taken into account, which makes it essential to know what type of patient would benefit from the system being designed.

A further design aspect to consider is what the system aims to improve in the patient, as this will determine the patient’s interaction with the system. Exergames are usually oriented toward improving walking abilities and body posture (both static and dynamic) [81].

### Software design criteria

The software contained in a gamified rehabilitation system must balance clinical rigor, playful stimulation, and technical quality. The main lines that should guide its conception are developed below.

#### Accessibility and usability

As seen in the general guidelines, the interface must adapt to the motor, visual, and cognitive deficits commonly found after a stroke. Linear navigation, high-contrast iconography, and resizable typography reduce the patient’s cognitive load and facilitate system comprehension [27]. In-system tutorials (both for the games and for device use) and an always accessible help module would avoid dependence on the therapist during sessions (both in the clinic and at home). Exploring redundant controls in the interface (touch, voice, or simple gestures) limits frustration arising from fine-motor failures. In practice, this means providing multiple alternative input methods for each basic action. For example, large on-screen buttons and broad arm movements can be used instead of the standard method. This allows patients with severe fine motor impairments to perform the exercises independently.

Performance history, fatigue, and aesthetic preferences are modeled in real time to adjust kinematic parameters (speed, range of motion) and game parameters (level, rate at which challenges appear). Functional personalization (for example, altering virtual gravity in a throwing game) increases the perception of competence, while aesthetic personalization (colors, avatar outfits) enhances autonomy [82]. Every adaptation must be transparent: showing the patient why the system modifies the task increases trust and reduces the sense of arbitrariness. This can be implemented with short on-screen messages or another visual feedback.

Visual feedback (accuracy meters, progress bars, or avatar reflections), auditory feedback (rhythmic sounds or sound effects aligned with the narrative), and, where appropriate, haptic feedback constitute the essential motorlearning loop [83]. Continuous correction indicators (for example, color codes) and brief summaries at session closure (post-game feedback) are recommended, avoiding overloading the pa- tient with technical metrics. When an error or failure oc- curs in the game, the software should gently guide the user toward correct execution instead of penalizing or abruptly stopping the experience.

#### Engagement and meaningful play

To sustain adherence, the software must ensure that each action has a clear effect integrated into the game narrative, thus generating meaningful play [84]. The balance between challenge and skill is maintained by automatically adjusting difficulty, while failure should be presented as an opportunity for learning, never as severe punishment, in order to protect the patient’s self-esteem [85]. Immediate visual or auditory rewards reinforce the sense of progress without saturating the user with superfluous stimuli.

Therefore, a proper understanding of game elements, their mechanics, and their dynamics is necessary to integrate them appropriately with the aesthetic component, as already seen in the MDA framework. It is also important to design an adaptive and emotionally safe immersive environment for the patient in order to achieve positive clinical outcomes [86].

#### Artificial intelligence (AI) and machine/deep learning

Artificial Intelligence (AI) algorithms add large-scale objectivity and personalization. Convolutional networks can transform inertial data series into automatic diagnostics of movement quality with *R*^2^coefficients close to 0.97 [56]. In adaptive control, *assist-as-needed *models regulate challenge intensity based on fatigue and progress predictions [28, 52, 87]. Recommendation systems also discern when to introduce new minigames or vary the narrative to prevent boredom. The key is to explain to the therapist (and, when pertinent, to the patient) the reasons for each adjustment, mitigating the perception of a “black box” [55].

Beyond control and recommendation algorithms, the integration of AI must encompass the intelligent fusion of heterogeneous **sensor **hardware. In [29] accelerometers, IMUs, flexion and pressure sensors, biosignals (EMG, MMG), and depth cameras are compared to automatically estimate clinical scores such as FMA or Wolf Motor Function Test (WMFT). Model-based approaches (Random Forest, SVM, CNN, LSTM) translate multimodal data into continuous values covering up to 73% of the FMA scale, whereas *model-free *methods (threshold counting, asymmetry indices) offer lightweight solutions at the cost of limited accuracy. Designing the software therefore involves choosing learning architectures that extract complex features while maintaining clinical interpretability. An AI designed in this way can convert raw readings from multiple sensors into objective, continuous evaluations requiring minimal effort from patients and therapists. This is also applicable to VR [62] and telerehabilitation [66]. 

In [88], a review was conducted on the use of artificial cognitive systems applied to the stimulation and rehabilitation of executive functions, highlighting the use of adaptive learning algorithms as an essential software design criteria.

#### Security, privacy, and data governance

Compliance with international standards in the design of software for medical environments is a fundamental aspect to ensure its safety, effectiveness, and traceability. The IEC 62,304 standard, which regulates the software lifecycle for medical devices, has been used as a foundation for structuring safer and more efficient software development processes. This standard has supported the creation of roadmaps for process improvement focused on structures risk management and requirements traceability throughout the software development cycle [89]. Furthermore, its application has extended to the design of immersive virtual reality systems used in home-based therapies highlighting its value in ensuring patient safety, system interoperability, and user trust in remote monitoring settings [90].

On the other hand, human-centered interaction is also frequently emphasized in the design of health-oriented software. In [91], a personal health record system for the management of metabolic syndrome is developed based on the principles of the ISO 9241− 210:2010 standard. It is essential to consider the needs, abilities, and limitations of end users form the early stages of design to enhance the software’s usability and acceptance across various clinical contexts.

Effective gamified rehabilitation software arises from the confluence of accessibility, meaningful gameplay experience, context-sensitive adaptation, and, potentially, AI algorithms. With these guidelines, developers can transform therapeutic repetition into an interactive adventure that maintains motivation and, above all, yields measurable functional gains in post-stroke patients.

## Future work

This section presents the future research lines most commonly found in the literature. The main theoretical frameworks for gamified design are then discussed. Finally, a development and clinical-iteration methodology is proposed based on the review. 

Among the main future directions, the following have been identified:

1. Need for multidisciplinary approaches throughout the design of gamified systems [28, 55, 58, 64, 68].
2. Longitudinal studies and larger samples in clinical re- search [27, 32, 52, 56, 58, 59, 62, 65, 67, 69, 77, 78, 80].
3. Studies examining the cost-effectiveness relationship of gamified systems applied to neurorehabilitation [26, 29, 59, 67, 69, 78, 79].
4. Design of systems that allow a multiplayer mode to encourage patients’ social participation [6, 64, 66, 79].
5. Systems that enable both in-clinic integration and tele- monitoring [26, 52, 53, 58, 65, 68, 70].
6. Design of modular, lightweight, and accessible systems [28, 32, 53, 69, 70, 77].
7. New clinical scales, metrics, and evaluation protocols adapted to the technologies and games, enabling comparative outcome studies [26, 27, 41, 54, 63, 66,77

As have seen, coverage of social and emotional domains is limited. This means that therapists receive little guidance on how to integrate gamified systems into clinical practice. Therefore, future research should, along these lines:

1. Systematically include emotional and social outcomes alongside motor and functional measures as primary and secondary measures.
2. Report in more detail on the clinical specific settings and contexts of patients using gamified systems, such as previous experience with video games, HEXAD player profiles, game preferences, and subjective perceptions of game elements, and how these factors influence patient gamified system interactions.
3. Test preliminary design solutions experimentally that directly address these underrepresented areas.

### Theoretical frameworks for gamified design

This section presents some of the most recurrent theoretical frameworks within which gamified design in healthcare contexts is usually framed. They will be briefly described in order to provide a generic vision. Something similar was presented by the authors of [71], where, based on the Game-Flow model and the User-Centered Design (UCD), they presented their own conceptual framework with three distinct phases: identification of the patient’s characteristics, development of a SG, and its subsequent evaluation.

Previously, the MDA framework has already been seen, through which the gamification domains have been unraveled and which has served to support the classification of the elements and components seen in the bibliography.

On the other hand, Flow Theory (a concept from positive psychology), developed by Mihály Csikszentmihalyi, considers flow as a fundamental element of enjoyment [71, 92–94]. The GameFlow model, based on this Flow Theory, provides criteria for video games to be fun and enjoyable [46, 71, 95]. The design elements considered are (1) focus,(2) challenge, (3) player skill, (4) control, (5) clear goals, (6) feedback, (7) immersion, and (8) social interaction.

According to the UCD, although there are different definitions, they all end up focusing on a key and common element: the design process is oriented towards the user’s needs [4, 71, 85, 96]. This makes it possible to design, among other things, gamified systems adapted to the preferences, wishes and needs of the users (in this case, stroke patients). Moreover, it usually consists of three phases: (1) analysis, (2) design, and (3) evaluation. It is usually accompanied by an iterative approach. For these reasons, it has been considered to take it into account for the development of the scales proposed here.

Then, Self-Determination Theory (SDT) identifies three basic psychological needs to promote well-being and intrinsic motivation [4, 5, 46, 47, 82, 93, 97, 98]: autonomy (feeling of control over one’s actions and decisions), competence (feeling of mastery and effectiveness over one’s actions), and relatedness (need to feel connected and to belong). Based on the SDT, HEXAD typology emerges for classifying players into different profiles, according to their preferences and motivations (intrinsic and extrinsic) [47, 99, 100, 102]. The six profiles found, along with their primary motivations, are as follows: (1) philanthropist (purpose), (2) socialiser (relationships), (3) free spirit (autonomy), (4) achiever (competence), (5) player (extrinsic rewards), and (6) disruptor (change).

### Personalized therapy

This section outlines how personalized therapy in gamified post-stroke rehabilitation can be advanced through clear, it- erative design criteria for both hardware and software.

Commercial consoles such as the Nintendo Wii and Sony PlayStation EyeToy have been explored in rehabilitation because they are inexpensive and widely available, yet their generic nature limits how well they address stroke-specific deficits [94]. Custom-made games, on the other hand, embed ecologically valid movements directly in the play context and can therefore target impairments more precisely [94]. Therefore, while commercial video game solutions offer a high level of gamification, they do not necessarily match the actual preferences and needs of patients. This reinforces the need for a truly patient-centered design in all areas: physi- cal, cognitive, emotional and also in terms of their intrinsic preferences for games.

Despite extensive literature on gamified interventions, a persistent gap is the limited personalization of game mechanics: users respond differently to identical elements, and generic designs can yield experiences that are irrelevant or minimally useful [16, 43, 103–105]. Selecting and tuning gamification techniques for clinical settings thus remains an open challenge [106]. Several reviews also underline the absence of standardized development protocols and a unified explanatory model for how gamification exerts its effects [2, 33, 107].

Although other works offered design frameworks for gamified therapy [109], consensus is still missing on a generic theory that can guide joint hardware-software development across diverse patient groups. Opinions differ on how complex those games should be: minimalist mechanics keep interactions manageable [110], yet excessive simplification risks degenerating into “pointification,” which fails to sustain engagement or achieve therapeutic goals [4, 5, 14, 16, 43].

Conversely, intricate designs can overwhelm some users, so the optimal strategy is stratified complexity enough richness to motivate but adaptable to individual cognitive and physical load. Standard “one-size-fits-all” approaches [104, 111] therefore need to be complemented by adaptive methodologies, yet direct comparisons between standardized and personalized gamification remain scarce [104].

Basing on the most recent literature (2019–2023) and analyses of contemporary systems, the present study distills actionable criteria that support an iterative, clinician informed workflow capable of evolving with patient expectations. By formalizing these criteria it pretends to offer a roadmap for engineers, game designers, and therapists who aim to develop comprehensive, patient-centric SG solutions for neurorehabilitation while maintaining affordability, accessibility, and clinical relevance.

## Discussion

This work offers an integrative overview of the evolution that gamification in post-stroke neurorehabilitation has undergone between 2019 and 2023. Based on a corpus of 68 self-developed prototypes reported in the literature, this section discusses (a) the main findings and their contribution with respect to previous reviews, (b) the design implications arising from the results, (c) the methodological limitations detected, and (d) the emerging lines of research.

### Synthesis of the findings

In line with objective **O1**, the analysis reveals a clear predominance of solutions oriented toward the *functional *domain (98.5%), with a marked bias toward motor recovery (79.4%). The affective (4.4%) and social (6.8%) domains remain under represented, confirming the trend noted by [16]. At the *setup* level, technological fragmentation persists: PCs and conventional screens still dominate (52.9%), whereas HMDs are consolidating (35.3%) as a gateway to VR/IVR environments. However, the convergence of several interfaces within the same device (e.g., VR + exergames or EMG+ VR) suggests a shift toward hybrid systems capable of adapting difficulty in real time, as said in [63]. Regarding software, Unity (51.5%) remains the de facto development engine, although the mean of 3.3 minigames per device evidences an effort to diversify the experience and mitigate fatigue.

### Contribution relative to the literature

Compared with previous reviews focused on isolated categories (robotics [26], mHealth [27], or sensors [29]) the present study integrates *hardware*, *software*, and *gamification criteria* within a single conceptual framework. This holistic approach makes it possible to detect under-exploited synergies: (a) combining upper-limb exoskeletons with EMG biofeedback to tune *assist-as-needed *support; (b) overlaying MR with Transcranial Direct Current Stimulation (tDCS) or FES to intensify plasticity; and (c) using AI to orchestrate multimodal data and personalize difficulty moment by moment, overcoming the “pointification” denounced in [4, 5, 14, 16, 43].

### Implications for the design of gamified systems

The eight generic hardware criteria (Sect. 6.1) and the proposed software guidelines provide an actionable *check-list* for multidisciplinary teams:

1. **Adaptive multimodal feedback**. Evidence indicates that visual + auditory + haptic *feedback*, synchronized with physiological metrics, maximizes motor learning and motivation.
2. **Sensor–robot–AI fusion**. Integrating accelerometers, EMG, and vision within intelligent control loops reduces therapist workload and calibrates assistance in real time.
3. **Meaningful gamification**. The MDA framework is useful, but incorporating other models (HEXAD, SDT) would allow mapping motivations and tailoring mechanics (intrinsic vs. extrinsic rewards) to specific patient profiles.
4. **Accessibility and costs**. Arduinos and microcontrollers prove to be key allies for low-cost prototypes, although ergonomics and robustness must be weighed against ready to use commercial devices.

### Study limitations

Like other reviews, the present work is constrained by the heterogeneity of the source studies: small sample sizes, disparate protocols, and scant reporting of standardized metrics limit the possibility of quantitative meta-analysis. In addition, the search was restricted to the 2019–2023 period and to self-developed prototypes, which may lead to conclusions biased with respect to a more detailed analysis of commercial solutions. Some bias may also have been introduced by not carrying out a systematic review using PRISMA. Finally, although the MDA framework guided the extraction of gamification components, alternative models exist whose use might have yielded additional nuances in the classification.

##  Conclusions

Between 2019 and 2023, research on gamified neurorehabilitation has been dominated by devices designed for functional purposes (98.5% of the 68 systems developed by the researchers themselves), with a clear bias towards motor recovery (79.4%), while cognitive rehabilitation (35.3%) andactivities of daily living (29.4%) receive comparatively less attention. Affective (4.4%) and social (6.8%) components remain marginal. Given this imbalance, future gamified systems should explicitly incorporate affective and social objectives as basic design requirements. This implies (i) systematically including emotional and social outcomes along-side motor and functional measures in clinical studies, and (ii) incorporating specific features, such as multimodal feedback and cooperative or competitive modes, to address motivation, mood and social engagement.

On the technological side, the analysis shows a rapid evolution towards hybrid configurations. Alongside conventional personal computers (52.9%), 35.3% of prototypes integrate HMD for IVR games, while sensors (32.4%), tangible interfaces (16.2%) and microcontroller-based solutions (19.1%) are increasingly combined, often with AI algorithms for assist-as-needed control. At the implementation level, 82.3% of systems are used exclusively in clinical settings, while only 10.3% are used at home and 8.8% use telerehabilitation, with only eight prototypes offering combined configurations. Given this distribution, new developments should prioritize lightweight, modular hardware that can be configured for use in clinics, homes and/or telerehabilitation. Since many current systems already collect kinematic, EMG and performance data, upcoming designs should exploit this multimodal information to implement real-time adaptation of difficulty and feedback, reducing therapist workload and enabling more precise personalization.

Regarding game design, almost all systems implement feedback mechanisms (98.5%), predominantly visual (94.0%), while time constraints appear in 45.6% of prototypes and point-based rewards in 60.3%. In contrast, multiplayer modes appear in only 4.4% of designs, and social dynamics remain rare. This pattern indicates a strong reliance on simple, extrinsic feedback with limited use of social and affective mechanisms. Therefore, beyond visual and point-based rewards, it is recommended to systematically map game mechanisms to motivational models such as Self-Determination Theory and HEXAD, and to explicitly report these mappings so that their impact on long-term adherence and engagement can be evaluated.

The eight cross-cutting hardware criteria identified in this study (usability, autonomy, safety, intensive use, ergonomics, cost/accessibility, objective evaluation and privacy), together with the proposed software guidelines, are based on the configurations actually observed in the 68 prototypes, where more than half of the systems are PC-based (52.9%) and almost a fifth rely on microcontrollers (19.1%), often without high-end robotics or HMDs. In this context, these criteria can be used as a practical checklist when selecting or designing gamified devices, especially in centers with limited resources. The studies reviewed here show that, when combined with well-designed mechanics and feedback, low-cost platforms can support clinically meaningful therapies, provided that usability, robustness and safety are carefully addressed.

Finally, the analysis suggests that relying solely on the MDA framework may be insufficient to guide long-term engagement and personalization in clinical practice. The proposed coding of mechanics, dynamics and aesthetics, together with the observed underrepresentation of affective and social domains and the predominance of single-player, point-based designs, indicates the need to integrate MDA with motivational theories such as Self-Determination Theory and HEXAD in the early stages of hardware–software codesign. In summary, to unlock the full potential of gamified neurorehabilitation, future systems should (1) systematically include affective and social design goals alongside motor and cognitive ones, (2) adopt modular hybrid hardware combined with AI-driven adaptation in clinical and home settings, and (3) rely on manageable data infrastructures and standardized evaluation protocols. The framework and criteria proposed in this article aim to translate these evidence-based trends into prioritized and actionable recommendations for engineers, game designers and therapists who wish to develop accessible, motivating and clinically effective gamified devices for stroke rehabilitation.

## Supplementary Information

Below is the link to the electronic supplementary material.

ESM 1 Supplementary Material 1 (PDF 108 KB)

ESM 2 Supplementary Material 1 (PDF 148 KB

## Glossary

10-MWT: 10-Meter Walk Test
6-MWT: 6-Minute Walking Test
ADL: Activities of Daily Living
AI: Artificial Intelligence
AMAT: Arm Motor Ability Test
AoU: Amount of Use
AR: Augmented Reality
ARAT: Action Research Arm Test
BBS: Berg Balance Scale
BBT: Box and Block Test
BCI: Brain–Computer Interface
BDI: Beck Depression Inventory
BESTest: Balance Evaluation Systems Test
BI: Barthel Index
BREQ: Behaviour Regulation Exercise Questionnaire
DASH: Disabilities of the Arm, Shoulder and Hand
DASS-21: Depression Anxiety Stress Scale
ECG: Electrocardiogram
EEG: Electroencephalogram
EMG: Electromyography
EQ-5D: European Quality of Life Five Dimensions
F8W: Figure of 8 Walk Test
FAB: Frontal Assessment Battery
FES: Functional Electrical Stimulation
FGA: Functional Gait Assessment
FIM: Functional Independence Measure
FMA: Fugl-Meyer Assessment
FSR: Force-Sensing Resistor
GAD-7: Generalized Anxiety Disorder 7-item
GDS-30: Geriatric Depression Scale
GEQ: Game Experience Questionnaire
GUI: Graphical User Interface
HADS: Hospital Anxiety and Depression Scale
HMD: Head-Mounted Display
HRQoL: Health-Related Quality of Life
IADL: Instrumental Activities of Daily Living Scale
IMI: Intrinsic Motivation Inventory
IMU: Inertial Measurement Unit
IPQ: Igroup Presence Questionnaire
IVR: Inmersive Virtual Reality
MAL: Motor Activity Log
MBEST: Mini Balance Evaluation Systems Test
MDA: Mechanics-Dynamics-Aesthetics
meCUE: Modular Evaluation of Key Components of User Experience
MI: Motricity Index
MMG: Magnetomyography
MoCA: Montreal Cognitive Assessment
NASA-TLX: NASA Task Load Index
Neuro-QoL: Quality of Life in Neurological Disorders
NMES: Neuromuscular Electrical Stimulation
NPC: Non-Playable Character
PAES: Physical Activity Enjoyment Scale
PGTQ: Perception of Game Training Questionnaire
PHQ: Patient Health Questionnaire
PPS: Pittsburgh Participation Scale
PvE: Player versus Environment
QoL: Quality of Life
QoM: Quality of Movement
QUEST: Quebec User Evaluation of Satisfaction with assistive Technology
RCI: Revised Competitiveness Index
SAQOL-39g: Stroke and Aphasia Quality of Life-39 generic
SDT: Self-Determination Theory
SF: Short Form Health Survey
SG: Serious Games
SIS: Stroke Impact Scale
SS-QOL: Stroke Specific Quality of Life Scale
SSQ: Simulator Sickness Questionnaire
SUS: System Usability Scale
SVG: Serious Virtual Games
TAM: Technology Acceptance Model
tDCS: Transcranial Direct Current Stimulation
TSRQ: Treatment Self-Regulation Questionnaire
TUG: Timed Up and Go test
UCD: User-Centered Design
UEQ: User Experience Questionnaire
UL: Upper Limb
USEQ: User Satisfaction Evaluation Questionnaire
USER-P: Utrecht Scale for Evaluation of Rehabilitation-Participation
VAS: Visual Analog Scale
VEQ: Virtual Embodiment Questionnaire
VR: Virtual Reality
VUE-Q: Virtual User Experience Questionnaire
WEMWBS: Warwick-Edinburgh Mental Wellbeing Scale
WHODAS: World Health Organization Disability Assessment Schedule
WHOQOL: World Health Organization Quality of-Life Scale
WMFT: Wolf Motor Function Test
