# Supplementary material for: Hardware and software design trends and considerations in gamified stroke rehabilitation: Results from a systematic review
Source: Med Biol Eng Comput. 2026 Mar 3;64(4):1203–30. doi: 10.1007/s11517-026-03518-y (PMC13121546; doi:10.1007/s11517-026-03518-y)
Supplement: Supplementary file 1 — Supplementary Material 1 (PDF 108 KB) [file 11517_2026_3518_MOESM1_ESM.pdf]

Medical & Biological Engineering & Computing manuscript No.
(will be inserted by the editor)

Hardware and software design trends and considerations in gamified stroke rehabilitation

Supplementary Material 1: game elements review

Juan J. Sánchez-Gil[a,1], **Aurora Sáez Manzano**[1], **Juan José Ochoa-Sepúlveda**[3], **Laura Muñoz-Millán** [1], **David Cáceres-Gómez** [3], **Rafael López-Luque** [2], **Eduardo Cañete-Carmona** [1]

[1]Departamento de Ingeniería Electrónica y de Computadores, Universidad de Córdoba, Edificio Leonardo Da Vinci, Campus de Rabanales, Córdoba, 14071, España
[2]Departamento de Ciencia de la Computación e Inteligencia Artificial, Universidad de Córdoba, Edificio Marie Curie, Campus de Rabanales, Córdoba, 14071, España
[3]Instituto de Neurociencias, Hospital Cruz Roja, P.º de la Victoria, Córdoba, 14004, España
Received: date / Accepted: date

Caption

In this supplementary material, a broad and detailed description is provided of the game design elements, focusing primarily on dynamics, mechanics, and aesthetics. Specifically, Table 1 lists all of these design elements. The aim is to offer a comprehensive review of the key components that make up serious games used in gamified neurorehabilitation therapies, with a particular emphasis on stroke. These elements are cited in Section 3.2.2 *Software* of **Chapter 3 – Key Gamification Parameters** in the main manuscript. The statistical analysis appears in **Chapter 4 – Current Trend Analysis**, also in the main manuscript.

[a]e-mail: sanchezgil@uco.es

Table 1: Classification of games design elements

Design Elements	Description	Refs.
Difficulty	The degree of challenge or effort required by the player to complete tasks within the game. It is a fundamental mechanic that guides the player's actions and adjusts the level of effort required to progress.	[1–6]
Voting	A system where players can cast votes to make collective decisions within the game that affect events or outcomes. Voting is a mechanism that establishes rules for how decisions are made in the game, which directly affects player actions. It is a system of interaction that guides the decisions and active participation of the players.	[7, 8]
Goals, tasks, challenges	Objectives that the player must complete in order to advance in the game, improve certain skills or receive rewards. They establish clear actions about what is required to progress and are fundamental to guiding the interaction.	[1, 2, 6–15]
Feedback	Real-time or post-game communication with the player/patient about their performance, success, or progress within the game. Feedback is a key mechanic that guides the player's actions and allows him to adjust his behavior based on the results he gets. It sets the rules for how the player's performance is evaluated.	[2, 6, 9, 11–25]
Unlocks	New skills, levels, items or features that are unlocked as the player progresses through the game. Unlocks are systems that reward the player's progress and change the game as they are earned. They act as mechanisms that condition the rate of progression and change the way you interact with the game.	[7, 8, 18]
Rewards	A benefit given to a player for completing a task or achieving a goal in the game. Rewards are bonus systems that guide player behavior, directly influencing motivation and the way challenges are approached. They are an essential mechanism for reinforcing desired behaviors in therapy.	[8, 9, 11, 12, 14, 18, 19, 21, 26]
Speed	The speed at which the player completes tasks or challenges in the game, which may affect their score or progress. Speed is a mechanic that conditions how the player must act under pressure of time or efficiency. It is a rule that defines the pace of the game and affects the outcome depending on how quickly the objectives are completed.	[1, 27]
Time	The amount of time the player has to complete a task or game session. It can also be entered as the time spent in the game. Like speed, it affects the pressure with which the player must complete objectives, but time is a global metric, while speed measures the alacrity with which a specific task is performed.	[1, 7, 8, 12, 13, 28]
Lifes	Number of chances the player has before failing or losing a quest or game session.	[1, 7]

Table 1: *(continued)*

**Design Elements**	**Description**	**Refs.**
Rules	A set of conditions that define how the game is played and what is allowed or prohibited. They are the framework within which the player interacts with the game, dictating what he/she can do and how game events unfold.	[13]
Help	Assistance or instructions provided to the player when they are having difficulty completing a task or encountering an obstacle. It often takes the form of suggestions, instructions, hints, or direct help that modifies the game to facilitate progress. It is a system that adapts the interaction to the player's performance.	[1]
Adaptability	The ability of a game to dynamically adapt to the player's needs or abilities by changing difficulty, content, or mechanics in real time. Adaptability affects the overall behavior of the game in response to player performance.	[1–3, 3, 13, 15, 16, 21, 24, 26, 27, 29–32]
Progress	A measure of player improvement through the game, reflecting task completion, level completion or skill improvement.	[7, 9, 11, 12, 14, 17, 19, 23]
Simplicity	The clarity and ease of use of the game design, ensuring that interactions and objectives are understandable to the player. It is an underlying force that conditions how the player perceives and experiences the game, facilitate participation without generating potential frustration.	[3, 16, 26]
Game mode	The different ways players can participate in the game, either individually, in groups, or in direct competition. It determines the type of emergent behavior (competition, cooperation, self-improvement) and influence motivation and engagement.	[6–9, 12, 20, 28, 33]
Chat rooms	Spaces where players can communicate in real time, facilitating social interaction and information sharing.	[7]
Story, narrative	The narrative context that structures the game and provides purpose and motivation to move forward. It is not a rule, but a force that gives meaning to tasks and encourages player engagement.	[7–12, 17, 28]
Leaderboards	They show the performance of players compared to others, encouraging competition.	[7–9, 11–14, 18, 21]
Score, Points	Bonuses obtained by the player as a result of completing actions or challenges within the game.	[1, 7, 10–13, 18, 21, 27, 28]
Levels	Stages or phases within the game that the player must complete, usually organized in order of increasing difficulty or complexity.	[7–9, 11–14, 18, 21]

Table 1: *(continued)*

Design Elements	Description	Refs.
Awards	Special recognition, such as medals or trophies, awarded to the player for outstanding achievements or important milestones in the game.	[7, 8, 11, 21]
Badges	Badges are tangible items that players receive as visible recognition of their achievements. They are earned by completing tasks or achieving certain in-game achievements. They are similar to the awards already described, but badges tend to be more common and are used to mark more specific or secondary milestones compared to awards, which tend to represent major achievements.	[1, 7, 9, 11, 12, 14, 18, 21]
NPC	Non-Playable Character (NPC) is game character controlled by artificial intelligence that interacts with the player, either to provide information or help, or to act as an opponent or companion. They are tangible characters in the game that the player sees and interacts with, but does not directly control. While they may affect the dynamics and mechanics of the game (e.g., by providing challenges or help), they are themselves components of the game environment. They interact with the rules, but do not dictate them, which positions them as elements of the game experience.	[5]
Avatars	Visual representations of the player or patient in the game environment. They add immersion and personalization.	[7, 9, 11, 12, 14, 18, 28]
Customization	Ability to customize or modify aspects of the game, such as the avatar, environment, or tools, to suit the player/patient's preferences. Customization is a feature that affects the tangible and intangible elements of the game, allowing players to configure their visual or interactive experience without changing the basic rules of the game.	[4, 7, 8, 14, 18]

References

1. Baranyi, R.: Deapsea: Workflow-supported serious game design for stroke rehabilitation. International Journal of Computer Games Technology **2023**, 1–15 (2023) https://doi.org/10.1155/2023/3169262
2. Mubin, O., Alnajjar, F., Mahmud, A., Jishtu, N., Alsinglawi, B.: Exploring serious games for stroke rehabilitation: a scoping review. Disability and Rehabilitation: Assistive Technology **17**, 1–7 (2020) https://doi.org/10.1080/17483107.2020.1768309
3. Li, L., Fu, Q., Tyson, S., Preston, N., Weightman, A.: A scoping review of design requirements for a home-based upper limb rehabilitation robot for stroke. Topics in Stroke Rehabilitation **29**(6), 449–463 (2022) https://doi.org/10.1080/10749357.2021.1943797
4. Cuthbert, R., Turkay, S., Brown, R.: The effects of customisation on player experiences and motivation in a virtual reality game. In: OZCHI'19: 31ST AUSTRALIAN CONFERENCE ON HUMAN-COMPUTER-INTERACTION, pp. 221–232 (2019). https://doi.org/10.1145/3369457.3369475
5. Alankus, G., Lazar, A., May, M., Kelleher, C.L.: Towards customizable games for stroke rehabilitation. Proceedings of the SIGCHI Conference on Human Factors in Computing Systems (2010)
6. Barrett, N., Swain, I., Gatzidis, C., Mecheraoui, C.: The use and effect of video game design theory in the creation of game-based systems for upper limb stroke rehabilitation. Journal of Rehabilitation and Assistive Technologies Engineering **3**, 2055668316643644 (2016) https://doi.org/10.1177/2055668316643644
7. Dykens, I.T., Wetzel, A., Dorton, S.L., Batchelor, E.: Towards a unified model of gamification and motivation. In: Sottilare, R.A., Schwarz, J. (eds.) Adaptive Instructional Systems. Design and Evaluation. HCII 2021. Lecture Notes in Computer Science, vol. 12792. Springer, ??? (2021). https://doi.org/10.1007/978-3-030-77857-6_4
8. Klock, A.C.T., Gasparini, I., Pimenta, M.S., Hamari, J.: Tailored gamification: A review of literature. International Journal of Human-Computer Studies **144**, 102495 (2020) https://doi.org/10.1016/j.ijhcs.2020.102495
9. Johnson, D., Deterding, S., Kuhn, K.-A., Staneva, A., Stoyanov, S., Hides, L.: Gamification for health and wellbeing: A systematic review of the literature. Internet Interventions **6**, 89–106 (2016) https://doi.org/10.1016/j.invent.2016.10.002
10. Ibrahim, E.N.M., Jamali, N., Suhaimi, A.I.H.: Exploring gamification design elements for mental health support. International Journal of Advanced Technology and Engineering Exploration **8**(74) (2021) https://doi.org/10.19101/IJATEE.2020.S1762123
11. Tuah, N.M., Ahmedy, F., Gani, A., Yong, L.N.: A survey on gamification for health rehabilitation care: Applications, opportunities, and open challenges. Information **12**(2) (2021) https://doi.org/10.3390/info12020091
12. Schöbel, S.M., Janson, A., Söllner, M.: Capturing the complexity of gamification elements: a holistic approach for analysing existing and deriving novel gamification designs. European Journal of Information Systems **29**(6), 641–668 (2020) https://doi.org/10.1080/0960085X.2020.1796531
13. Ferreira, B., Menezes, P.: Gamifying motor rehabilitation therapies: Challenges and opportunities of immersive technologies. Information **11**(2) (2020) https://doi.org/10.3390/info11020088
14. Mohd Zuki, F.S., Sulaiman, S., Rambli, D.R.A., Merienne, F., Mohamad Saad, M.N.: Sensory feedback and interactivity: Enhancing motivation and engagement for vr stroke rehabilitation. In: 2021 International Conference on Computer 'I&' Information Sciences (ICCOINS), pp. 333–338 (2021). https://doi.org/10.1109/ICCOINS49721.2021.9497200
15. Burke, J.W., McNeill, M.D.J., Charles, D.K., *et al.*: Optimising engagement for stroke rehabilitation using serious games. The Visual Computer **25**, 1085–1099 (2009) https://doi.org/10.1007/s00371-009-0387-4
16. Omar, M.Y.B., Rambli, D.R.A., Sulaiman, S., Shiratuddin, M.F., Merienne, F., Vanhalle, D.: Proposed conceptual design model of persuasive game for upper limb for stroke rehabilitation. In: 2019 IEEE Conference on Graphics and Media (GAME), pp. 44–48 (2019). https://doi.org/10.1109/GAME47560.2019.8980789
17. Huang, X., Xiang, X., Liu, Y., Wang, Z., Jiang, Z., Huang, L.: The use of gamification in the self-management of patients with chronic diseases: Scoping review. JMIR Serious Games **11**, 39019 (2023) https://doi.org/10.2196/39019
18. Al-Rayes, S., Al Yaqoub, F.A., *et al.*: Gaming elements, applications, and challenges of gamification in healthcare. Informatics in Medicine Unlocked **31**, 100974 (2022) https://doi.org/10.1016/j.imu.2022.100974
19. Ayed, I., Ghazel, A., Jaume-i-Capó, A., Moyà-Alcover, G., Varona, J., Martínez-Bueso, P.: Vision-based serious games and virtual reality systems for motor rehabilitation: A review geared toward a research methodology. International Journal of Medical Informatics **131**, 103909 (2019) https://doi.org/10.1016/j.ijmedinf.2019.06.016
20. Thomson, K., Todhunter-Brown, A., Bugge, C., Brady, M.: Commercial gaming devices for stroke upper limb

rehabilitation: The stroke survivor experience. Journal of Rehabilitation and Assistive Technologies Engineering **7**, 205566832091538 (2020) https://doi.org/10.1177/2055668320915381
21. Seaborn, K., Fels, D.I.: Gamification in theory and action: A survey. International Journal of Human-Computer Studies **74**, 14–31 (2015) https://doi.org/10.1016/j.ijhcs.2014.09.006
22. Darekar, A., McFadyen, B.J., Lamontagne, A., Fung, J.: Efficacy of virtual reality-based intervention on balance and mobility disorders post-stroke: a scoping review. Journal of NeuroEngineering and Rehabilitation **12**, 46 (2015) https://doi.org/10.1186/s12984-015-0035-3
23. Luque-Moreno, C., Ferragut-Garcías, A., Rodríguez-Blanco, C., Heredia-Rizo, A.M., Oliva-Pascual-Vaca, J., Kiper, P., Oliva-Pascual-Vaca, : A decade of progress using virtual reality for poststroke lower extremity rehabilitation: Systematic review of the intervention methods. Biomedical Research International, 342529 (2015) https://doi.org/10.1155/2015/342529
24. Staiano, A.E., Flynn, R.: Therapeutic uses of active videogames: A systematic review. Games for Health Journal **3**(6), 351–365 (2014) https://doi.org/10.1089/g4h.2013.0100
25. Saposnik, G., Levin, M., Stroke Outcome Research Canada (SORCan) Working Group: Virtual reality in stroke rehabilitation: A meta-analysis and implications for clinicians. Stroke **42**(5), 1380–1386 (2011) https://doi.org/10.1161/STROKEAHA.110.605451 https://www.ahajournals.org/doi/pdf/10.1161/STROKEAHA.110.605451
26. Korn, O., Tietz, S.: Strategies for playful design when gamifying rehabilitation. a study on user experience. PETRA ’17: 10th Int. Conference on PErvasive Technologies Related to Assistive Environments (2017) https://doi.org/10.1145/3056540.3056550
27. Grudpan, S., *et al.*: Virtual reality games for stroke rehabilitation: A feasibility study. In: Nunes, N.J., Ma, L., Wang, M., Correia, N., Pan, Z. (eds.) Entertainment Computing – ICEC 2020. Lecture Notes in Computer Science, vol. 12523. Springer, ??? (2020). https://doi.org/10.1007/978-3-030-65736-9_15
28. Ferreira-Brito, F., *et al.*: Game-based interventions for neuropsychological assessment, training and rehabilitation: Which game-elements to use? a systematic review. Journal of Biomedical Informatics **98**, 103287 (2019) https://doi.org/10.1016/j.jbi.2019.103287
29. Damaševičius, R., Maskeliūnas, R., Blažauskas, T.: Serious games and gamification in healthcare: A meta-review. Information **14**(2), 105 (2023)
30. Tosto, J., Tabacof, L., Herrera, J., Breyman, E., Dewil, S., Cortes, M., Correa-esnard, L., Kellner, C., Dangayach, N., Putrino, D.: Gamified neurorehabilitation strategies for post-stroke motor recovery: Challenges and advantages. Current Neurology and Neuroscience Reports **22** (2022) https://doi.org/10.1007/s11910-022-01181-y
31. Rodrigues, L.F., *et al.*: Personalized gamification: A literature review of outcomes, experiments, and approaches. In: Proceedings of the International Symposium on Gamification and Games for Learning (GamiLearn’20), p. 8. ACM, ??? (2020). https://doi.org/https://doi.org/to-bedefined
32. Charles, D., Holmes, D., Charles, T., McDonough, S.: Virtual reality design for stroke rehabilitation. In: Rea, P. (ed.) Biomedical Visualisation. Advances in Experimental Medicine and Biology, vol. 1235. Springer, ??? (2020). https://doi.org/10.1007/978-3-030-37639-0_4
33. Vieira, C., Silva Pais-Vieira, C., Novais, J., Perrotta, A.: Serious game design and clinical improvement in physical rehabilitation: Systematic review. JMIR Serious Games **9**(3), 20066 (2021) https://doi.org/10.2196/20066
