# Supplementary material for: Hardware and software design trends and considerations in gamified stroke rehabilitation: Results from a systematic review
Source: Med Biol Eng Comput. 2026 Mar 3;64(4):1203–30. doi: 10.1007/s11517-026-03518-y (PMC13121546; doi:10.1007/s11517-026-03518-y)
Supplement: Supplementary file 2 — Supplementary Material 1 (PDF 148 KB [file 11517_2026_3518_MOESM2_ESM.pdf]

Medical & Biological Engineering & Computing manuscript No.
(will be inserted by the editor)

Hardware and software design trends and considerations in gamified stroke rehabilitation

Supplementary Material 2: complete analysis of the software and hardware used in new gamified systems for post-stroke therapy.

Juan J. Sánchez-Gil[a,1], Aurora Sáez Manzano[1], Juan José Ochoa-Sepúlveda[3], Laura Muñoz-Millán [1], David Cáceres-Gómez [3], Rafael López-Luque [2], Eduardo Cañete-Carmona [1]

[1]Departamento de Ingeniería Electrónica y de Computadores, Universidad de Córdoba, Edificio Leonardo Da Vinci, Campus de Rabanales, Córdoba, 14071, España
[2]Departamento de Ciencia de la Computación e Inteligencia Artificial, Universidad de Córdoba, Edificio Marie Curie, Campus de Rabanales, Córdoba, 14071, España
[3]Instituto de Neurociencias, Hospital Cruz Roja, P.º de la Victoria, Córdoba, 14004, España
Received: date / Accepted: date

Caption

In this supplementary material, an exhaustive analysis of the hardware and software used in the new designs reviewed in [1] is provided. This complete analysis is presented in Table 1, offering all the observed trends in great detail. These trends are then statistically examined in **Chapter 4 – Current Trends Analysis** of the main manuscript.

[a]e-mail: sanchezgil@uco.es

Table 1:
Classification of new trends in gamified stroke and neurorehabilitation design

Ref.	Game Env.	No. games	Technology (Hardware)	Development Software	Patient Domain (Implementation)	Gamification Components		
						Mechanics	Dynamics	Aesthetics
[2]	SVG,VR	10 games	Tangible controller, own BrightBrainer grip, HTC Tracker, HTC VIVE IR, PC, screen, desk and security system	Own software delevopment	Functional: Motor, cognitive (clinic)	Speed, Difficulty, Feedback (visual), Rules, Challenges/Objectives, Help, Time (countdown), Rewards	Simplicity, Progress, Game Mode (single-player), Adaptability (performance)	Score (scoring and penalties), Avatar, Levels, Aesthetics (3D, first person)
[3]	IVR	Virtual Environment	NMES, Robotic glove (Arduino MEGA, servomotors), BCI, Kinect and Leap Motion, HMD Oculus Rift, PC	Unity, Make Human software, GUI	Functional: Motor (clinic)	Feedback (visual, sound, FES, haptic), Goals, Rules, Help	Game Mode (single-player), Progress	Avatars, NPC (assistant), Aesthetic (3D, simple, first person), Customization
[4]	VR	Virtual Environment	BCI recoveriX system, g.USBamp (g.tec medical engineering GmbH), PC	Not Espacified	Functional: Motor, cognitive (clinic)	Feedback (visual, sound, FES), Task, Rules, Help (instructions)	Game Mode (single-player), Progress	Avatar, Aesthetic (3D, simple, first person)
[5]	SVG	1 game	Tangible controller, Load cell, INA125 instrumentation amplifier, PC, projector, Atmel's 32-bit ARM Cortex-M3 processor	Not Specified, GUI	Functional: Motor (clinic)	Feedback (visual), Object Behavior (impacts), Rules, Difficulties, Rewards	Progress, Customization, Game Mode (singleplayer), Simplicity	Points, Aesthetics (classical games, 2D, simple)
[6]	SVG	6 games	Tablet Samsung Galaxy Note Pro	Not Specified, GUI	Functional: Cognitive (Clinic, In-Home)	Feedback (visual, sounds), Difficulties, Rules, Goals, Time (countdown), Speed	Simplicity, Game Mode (singleplayer), Progress	Aesthetic (3D shapes), Levels, NPC (neutrals)
[7]	SVG	1 game	EMG sensors: MAN-012-2-6 (Delsys Inc.), MPL115A2 barometric pressure sensors (Freescale Semiconductor Inc.), 9-axis IMUs (MTw Awinda, Xsens Inc.)	MATLAB, Pygame Library	Functional: Motor, ADL (clinic)	Feedback (visual, sound), Goals, Time (countdown), Help, Rules, Rewards	Game Mode (single-player)	Points, Aesthetic (2D, simple), Levels
[8]	Tangible SG	7 games	Tangible elements, RFID sensors, desk, Arduino MEGA, Pepper Robot (Softbank Robotics Aldebaran), PC, screen	Not Applicable	Social, Functional: Motor, cognitive, ADL (clinic)	Feedback (visual, sound), Goals, Difficulties, Help (instructions), Time (limited or unlimited), Rewards, Rules	Game Mode (single-player, with social robot), Progress	Points, Levels
[9]	Tangible SG, SVG	8 games	LCD screen multi-touch, desk	Not Specified	Social, Functional: cognitive (clinic)	Feedback (visual), Goals, Difficulties, Rules, Time (countdown), Speed, Rewards	Game Mode (individual and team competition), Chat Rooms	Awards (trophies), Leaderboards, Avatar, Aesthetics (2D, sports)

Table 1: *(continued)*

Ref.	Game Env.	No. games	Technology (Hardware)	Development Software	Patient Domain (Implementation)	Gamification Components		
						Mechanics	Dynamics	Aesthetics
[10]	Tangible SVG	1 game	Tangible elements, touch screen	Not Specified, GUI	Functional: Motor (clinic)	Feedback (visual), Time (countdown), Help (instructions), Rules, Goals, Rewards, Difficulties	Game Mode (single-player), Adaptability (game parameters), Story	Points, Aesthetic (3D), NPC
[11]	SVG	1 game	Tangible elements, LED TV screen and a sensitive multi-touch infrared layer, PC, desk	Not Specified	Social, Functional: Motor (clinic)	Feedback (visual), Time (Countdown), Rewards, Rules, Goals	Game Mode (multi-player: collaborative, co-active and competitive), Simplicity, Chat Rooms	Points, Leaderboards, Aesthetic (2D, simple)
[12]	VR	8 games	Kinect	Scratch 2.0	Functional: Motor (clinic)	Feedback (visual, sound), Rewards, Goals, Rules, Difficulties	Game Mode (single-player), simplicity	Aesthetic (2D, simple), NPC (hostile, neutrals)
[13]	SVG	1 game	Self-designed wearable EMG	Python-based gaming platform, Blender	Functional: Motor (in-home)	Feedback (visual, sound), Rewards, Rules, Difficulties, Time, Goals, Help (instructions)	Game Mode (single-player), Adaptability (performance), Progress	Points (scoring and penalties), Aesthetics (2D, 3D, simple), Customization
[14]	SVG	Virtual Environment	PC	LIVE©, ARA-Virtual Heroes Go framework, GUI	Social (telerehabilitation)	Feedback (visual, sound), Rewards, Rules, Help (assistance)	Game Mode (interactive), Chat Rooms	Points, Leaderboards, NPC (neutrals), Aesthetic (3D), Customization, Avatar
[15, 16]	Tangible SVG, exergames	6 games	Multiple tangible accessories, sensors (magnetometer, accelerometer, gyroscope), velostat, smartphone, tablet	Not Specified	Functional: Motor (clinics, in-home)	Feedback (visual, sound), Rules, Rewards, Goals, Unlocks (songs)	Game Mode (single-player), simplicity	Points, Aesthetic (2D, simple)
[17]	IVR	Virtual Environment	OpenBCI EEG and EMG, HMD Oculus, PC, OpenBCI board, IMUs (Teensy 3.2 microcontroller and two LSM9DS0 9DOF sensors)	Unity, Oculus SDK	Functional: motor (clinic)	Feedback (neurological, visual, sound, haptic), Rules, Time (countdown), Rewards, Tasks	Game Mode (single-player)	Points, Aesthetics (3D, first person)
[18]	IVR	6 virtual environments	HMD Oculus Rift S, PC	Unity, Virtual Reality Toolkit, GUI	Functional: Cognitive, ADL (clinic)	Feedback (post game), Challenges, Rules, Object Behavior (grip), Help, Difficulties	Game Mode (single-player), Progress	Levels, Avatar, Customization, Aesthetic (3D, realistic, first person)
[19]	VR, exergames	Virtual World	Sensorized shoes (FSR), Fitness World3100 Motorized Treadmill, PC, projector, BioPac ECG MP150 (BioPac Systems Inc.)	Nos Specified, GUI	Functional: Motor (clinic)	Feedback (visual, sound), Rules, Rewards, Time (countdown), Speed, Tasks	Game Mode (single-player), Adaptability (performance)	Points, Avatar, Aesthetic (3D, third person)

Table 1: *(continued)*

Ref.	Game Env.	No. games	Technology (Hardware)	Development Software	Patient Domain (Implementation)	Gamification Components: Mechanics	Gamification Components: Dynamics	Gamification Components: Aesthetics
[20]	VR, exergames	Virtual World	Custom-built self-pace treadmill, screen, 6-camera Vicon™ (Vicon), Vicon Nexus™ 1.8.5 motion capture system	Maya LT™ 2016 (Autodesk), Pegasus Advanced™ (Vicon), Unreal Engine 4 (Epic Games)	Functional: Motor (clinic)	Feedback (visual), Rules, Help (instructions), Tasks	Game Mode (single-player)	Aesthetics (3D, realistic, diferents POV, third person)
[21]	VR	3 games	PC, Kinect	Not Specific	Functional: Motor (in-home)	Feedback (visual, post game), Rewards, Help (instructions), Rules, Difficulty, Time (countdown)	Game Mode (single-player), Progress	Points, Levels, Aesthetic (first/third person, 3D, realistics)
[22]	VR, IVR	Virtual Environment	HMD Oculus Rift DK2 and HMD HTC Vive, Xbox 360© wired controller, screen	Unity	Functional: ADL (clinic)	Tasks, Time (countdown), Rules	Game Mode (single-player)	Aesthetics (3D, realistic, first person)
[23]	IVR, exergames	6 minigames	X-erfit 4.000 Pro Runner treadmill, HMD HTC Vive, Qualisys AB 3D motion capture, emergency button	Unity, GUI	Functional: cognitive, motor (lab)	Feedback (visual), Rules, Rewards, Help, Challenges, Speed	Game Mode (single-player), Adaptability	Avatar, Points, Levels, Aesthetic (3D, first person)
[24]	VR, IVR	1 game	HMD HTC Vive Pro, PC	Unity	Functional: ADL (clinic)	Feedback (visual, sound, post game), Difficulty, Rewards, Help (instructions), Tasks, Rules	Game Mode (single-player), Adaptability (performance), Progress	Points, Aesthetic (3D, hyper-realistic, first person)
[25]	VR	4 games	PC, Leap Motion Controller	Unity	Functional: Motor, cognitive (clinic)	Feedback (visual, sound), Rewards, Tasks, Help (instructions), Rules, Time (countdown)	Game Mode (single-player), Adaptability (performance, avoidance of frustration)	Score, Avatar, Aesthetics (3D, first person)
[26]	VR	1 game	Myo IMUs (Thalmic Labs Inc.), screen	Not Specified	Functional: Motor (clinic)	Feedback (visual), Challenges, Help (instructions), Rules, Difficulty, Rewards, Object Behavior (size, accuracy), Lifes, Time (countdown), Speed	Game Mode (single-player), Adaptability (performance, ability)	Points (scoring and penalties), Avatar, Levels, Aesthetics (3D, realist)
[27]	VR	4 games	Kinect, PC	Unity, GUI	Functional: Motor, ADL (clinic)	Feedback (visual, sound), Rules, Rewards, Tasks, Time (countdown)	Game Mode (single-player), Adaptability (portability), Simplicity	Points (scoring and penalties), NPC (neutral), Avatar, Aesthetics (2D, simple; 3D, realist, first person)

Table 1: *(continued)*

Ref.	Game Env.	No. games	Technology (Hardware)	Development Software	Patient Domain (Implementation)	Gamification Components		
						**Mechanics**	**Dynamics**	**Aesthetics**
[28]	IVR	Virtual Environment	HMD Valve Index VR, HTC VIVE Tracker 2.0	Unity, SteamVR SDK	Functional: Motor (clinic)	Feedback (visual, haptic), Help (instructions), Rules	Game Mode (single-player, accompanied by the therapist), Simplicity	Avatar, Aesthetic (3D, third person)
[29]	IVR	2 games	HMD Oculus Quest	Not Specified, GUI	Functional: cognitive, ADL (clinic)	Feedback (visual, sound), Rules, Difficulty, Rewards, Goals, Time, Speed, Help (instructions)	Game Mode (single-player)	Points (scoring and penalties), Levels, Avatar, Aesthetics (3D, hyper-realistic, first person)
[30]	IVR	4 Virtual Environments	HMD Oculus Rift, Xbox 360 controller	OsiriX Software, Maya (Autodesk), Unity	Functional: cognitive (clinic)	Feedback (visual, sound), Rules, Help	Game Mode (group work), Narrative	Aesthetic (3D, hyper-realistic)
[31]	VR	Virtual Environment	Omega.7 haptic feedback device (Force Dimension Inc.), HMD Oculus Rift	Chai3D, OpenGL libraries	Functional: Motor	Feedback (haptic), Rewards, Challenges, Rules	Game Mode (singleplayer), Progress, Simplicity	Score, Aesthetic (3D)
[32]	VR	4 games	Touchscreen, PC, Bend Sensor (Images SI Inc.), Fingerless Mitten (Handana Corp.)	Unity, GUI	Functional: Motor (clinic, in-home)	Feedback (visual, FES), Rewards, Rules, Challenges, Difficulties, Time (countdown), Help (instructions), Speed	Game Mode (single-player), Adaptability (performance)	Points, Leaderboards, Levels, Aesthetics (2D, 3D, classic games)
[33]	VR	2 games	Inertial sensors (Shimmer3), Kinect, projector	Visual C#, MySQL (Oracle Corporation), Blender, GUI	Functional: Motor (clinic, telerehabilitation)	Feedback (visual, postgame), Rewards, Time (countdown), Rules, Tasks	Game Mode (single-player), Adaptability (manual), Simplicity, Progress	Points, Avatar, Aesthetic (3D)
[34]	VR	3 games	Kinect	Unity, DSS	Functional: Motor, cognitive (clinic, telerehabilitation)	Feedback (visual, postgame), Rewards, Rules, Difficulty, Time (spent)	Game Mode (single-player), Simplicity, Adaptability (manual), Progress	Points, Aesthetic (2D, 3D, classic games)
[35, 36]	VR	5 games	PC, web camera, GT Platform controller, FSR	Torque Game Engine (Garage Games), Markov Decision Process Engine (Perseus), OpenCV, GUI	Affective, Functional: Motor, ADL (clinic, telerehabilitation)	Feedback (visual), Difficulty, Help, Speed, Time (countdown), Challenges, Rules	Game Mode (single-player), Adaptability (performance)	Avatar, Levels, NPC (animals; hostiles), Aesthetic (2D; 3D, first person)

Table 1: *(continued)*

Ref.	Game Env.	No. games	Technology (Hardware)	Development Software	Patient Domain (Implementation)	Gamification Components		
						Mechanics	Dynamics	Aesthetics
[37]	VR	6 games	BCI-Assisted Soft Robotic Glove, PC, screen, EEG amplifier	Not Specified	Functional: Motor, ADL (clinic)	Feedback (visual), Tasks, Lifes, Rules, Object Behavior (touch, grip)	Game Mode (single-player)	Avatar, Aesthetic (3D, realistic, first person)
[38]	VR	4 games	Kinect, BWT901CL (accelerometer, inclinometer, bluetooth), JY901 module chip, PC	Unity	Functional: Motor, ADL (clinic, in-home)	Feedback (visual), Rewards, Challenges, Rules, Time (countdown)	Game Mode (single-player)	Points, Avatar, Aesthetic (3D)
[39]	SVG	2 games	Biopac MP150 (Bipac Systems Inc.), Own EMG-VR Handshake Module (Arduino MEGA), PC	Vizard Software (Worldviz Llc), Google Sketch Up	Functional: Motor (clinic)	Feedback (visual, sound), Rules, Rewards, Time (countdown), Challenges	Game Mode (single-player), Progress	Points, Aesthetics (3D)
[40]	IVR	1 game	HMD Meta Quest 2, C2-HDLF vibrotactors (Engineering Acoustics Inc.), NIR-Sport2 (NIRx Medical Technologies)	Unity	Functional: Motor (clinic)	Feedback (visual, sound, haptic), Rewards, Rules, Object Behavior (collision)	Game Mode (single-player)	Points, Avatar, Aesthetics (3D, music, rhythm)
[41]	SVG	6 games	Own exoskeleton	Not Specified, GUI	Functional: Motor, ADL	Feedback (visual), Rules, Help (assist), Tasks	Game Mode (single-player)	Aesthetic (2D, simple)
[42]	SVG	2 games	BCI device, Tobii EyeX2 eye tracker (Tobii)	Unity, OpenViBE	Affective, Functional: cognitive	Feedback (visual), Rules, Rewards, Goals, Object Behavior (collision)	Game Mode (single-player)	Points (scoring and penalties), Aesthetics (2D)
[43]	Tangible SG	1 game	Own hand orthosis, RehaMove Pro stimulator (Hasomed), MPU9250 IMU (TDK InvenSense), A201, A401 FSR (Tekscan Flexiforce), qIDmini R1170I RFID module, foam padded tangible objects	Not Applicable, GUI	Functional: Motor, ADL	Feedback (visual, FES), Tasks, Rules, Help (assistance)	Game Mode (single-player), Adaptability (manual), Simplicity	-
[44]	SVG, VR	2 games	Own 3 DOF robot (pneumatically actuated), screen	Not Specified	Functional: Motor (clinic)	Feedback (visual), Rewards, Tasks, Time, Rules, Help (assist)	Game Mode (single-player)	Points, Avatar, Aesthetics (2D)
[45]	VR	9 games	PlayStation Eye camera (Sony Computer Entertainment Inc.), PC, LCD screen, tangible elements	Unity, pattern tracking software (AnTS 2D-NUI)	Functional: Motor, cognitive, ADL (clinic)	Feedback (visual), Rewards, Difficulty, Tasks, Time, Rules, Help (instructions)	Game Mode (single-player), Adaptability (manual, performance, according to cognitive capacity)	Points, Avatar, Aesthetic (3D, realistic, first person)

Table 1: *(continued)*

Ref.	Game Env.	No. games	Technology (Hardware)	Development Software	Patient Domain (Implementation)	Gamification Components		
						**Mechanics**	**Dynamics**	**Aesthetics**
[46]	IVR	Virtual Environment	Leap Motion Camera, HMD Oculus CV1, PC, tablet	Unity, Orion SDK, Android Studio	Functional: Motor, cognitive (clinic)	Feedback (visual), Rules, Goals, Time	Game Mode (single-player)	Avatar, Levels, Aesthetic (3D, simple, first person)
[47]	VR	3 games	Kinect, PC, IMUs	Unity, C#, GUI	Functional: Motor, cognitive (clinic, telerehabilitation)	Feedback (visual, sound), Rules, Rewards, Help	Game Mode (Single/Multi player, competitive), Chat Rooms, Progress, Adaptability (Manual)	Points, Avatar, Levels, Aesthetic (3D, first and third person, ambience), Leaderboards, Awards (bonus), Customization
[48]	IVR, exergames	1 game	Mercury®med treadmill (h/p/cosmos sports), HMD HTC Vive RGB, PC, security system	Unreal Engine	Functional: Motor (clinic)	Feedback (visual, sound), Rules, Rewards, Speed, Goals, Unlocks	Game Mode (single-player), Adaptability (motor and cognitive abilities), Story, Progress	Points, NPC (pet), Customization, Aesthetic (3D, first person, music)
[49]	Tangible SG	3 games	Moto 360 SmartWatch (Motorola), tangible therapeutic objects, manometers, FSR, Raspberry Pi Zero Wireless platform, MPU9250, RFDuino platform	Android App	Functional: Motor, cognitive (clinic)	Feedback (postgame), Rules, Tasks	Game Mode (single-player), Simplicity, Progress	-
[50]	SVG	1 game	Triboelectric nanogenerator system, Op-Amp OP275, Arduino NANO, Bluetooth using HC-06, PC, wereable Controller	Python, MIT App Inventor	Functional: Motor (clinic)	Feedback (visual), Rules, Rewards, Goals, Lifes	Game Mode (single-player)	Points (scoring and buffs), Aesthetics (2D, classic games, sounds)
[51]	IVR	Virtual Environment	HMD HTC Vive Pro-Eye, PC	Unity	Functional: Motor, cognitive, ADL (clinic)	Feedback (visual), Rules, Difficulty, Help (instructions)	Game Mode (single-player), Simplicity, Story	Avatar, NPC (neutrals), Aesthetics (3D, realistic, first and third person, ambient sound)
[52]	AR	Virtual Environment	HMD HoloLens (Microsoft)	Unity, C#	Affective, Functional: Motor (clinic)	Feedback (visual, sound), Rules, Goals	Game Mode (single-player), Story	Aesthetic (3D, nostalgic)
[53]	VR, exergame	1 game	KineAssist (KA)-MX Robotic Treadmill, screen	Unreal Engine 4	Functional: Motor (clinic)	Leaderboards, Feedback (visual, sound), Difficulty, Rules, Time (countdown), Challenges, Speed	Game Mode (single-player, competitive, PvE)	Avatar, Levels, NPC (neutrals, hostiles), Aesthetic (3D, realistic)

Table 1: *(continued)*

Ref.	Game Env.	No. games	Technology (Hardware)	Development Software	Patient Domain (Implementation)	Gamification Components		
						Mechanics	Dynamics	Aesthetics
[54]	VR	Virtual Environment	PC, Leap Motion Controller	Unity, C#, Python, LMC SDK	Functional: Motor, cognitive (clinic)	Feedback (postgame), Rules, Rewards, Tasks, Time (spent), Object Behavior (grip)	Game Mode (single-player), Progress	Points, Avatar, Levels, Aesthetics (3D, first person)
[55]	IVR	1 game	HMD Oculus Quest 1	Unity, C#	Functional: Motor (clinic)	Feedback (visual, sound, haptic), Rules, Rewards, Tasks, Object Behavior (grip)	Game Mode (single-player), Simplicity	Points, Avatar, Aesthetic (3D, first person)
[56]	IVR, ex-ergames	Virtual Environment	MPU9250 IMU, ESP32 TTGo TTAudio 1.6 development board, HMD Oculus Rift, PC, Motomed Viva 2 trainer, SCIFITTM ISO7000 bike	Unity, Easy-Roads3D	Social, Functional: Motor (clinic)	Feedback (visual, sound), Rules	Game Mode (multi-player, collaborative), Chat Rooms	Avatar, Aesthetic (3D, hyper-realistic)
[57]	VR	Virtual Environment	Two BFS-U3–13Y3C-C cameras (FLIR Systems Inc.), PC	Unity, Open-Pose platform	Functional: Motor (clinic)	Feedback (visual), Rules, Rewards, Goals, Time, Lifes	Game Mode (single-player)	Points, Avatar, Aesthetic (3D, simple, first person)
[58]	SVG	Virtual Environment	IPad (Apple), PC	VRspace Pty Ltd, Auto-desk 3D Studio Max, Unity	Functional: Cognitive, ADL (in-home)	Feedback (visual), Rewards, Time (spent), Help (map), Rules, Tasks	Game Mode (single-player)	Points, NPC (neutrals), Aesthetic (3D, ambient sound)
[59]	IVR	5 games	HMD HTC Vive Pro	Unity	Functional: Motor, cognitive	Feedback (visual), Rules, Difficulty, Challenges, Help (instructions)	Game Mode (single-player)	Levels, Aesthetic (2D, 3D, first person)
[60]	SVG	Virtual Environment	PC	Unity	Functional: Cognitive, ADL (clinic)	Feedback (visual), Tasks, Rules, Help (instructions)	Game Mode (single-player), Simplicity	Aesthetic (3D, realistic)
[61]	VR, ex-ergames	Virtual Environment	Kinect	Microsoft Kinect SDK, Unity, GUI	Functional: Motor (clinic)	Feedback (visual, sound), Rules, Tasks, Help (video reference)	Game Mode (single-player), Adaptability (performance, manual), Progress	Avatar, Levels, Aesthetic (3D, third person)
[62, 63]	SVG	8 games	VNH2SP30 motor driver (2 actuators/30 A), Cable-Actuated Robot (1 y 2 DOF), Arduino MEGA, screen, PC, load cells, security system	MATLAB (MathWorks), Unity, GUI	Functional: Motor (clinic)	Feedback (visual, sound, haptic), Rewards, Goals, Rules, Difficulty	Game Mode (single-player), Simplicity, Progress, Adaptability (assistance), Narrative	Points, Levels, Aesthetics (2D, simple; 3D, realistic)

Table 1: *(continued)*

Ref.	Game Env.	No. games	Technology (Hardware)	Development Software	Patient Domain (Implementation)	Gamification Components: Mechanics	Gamification Components: Dynamics	Gamification Components: Aesthetics
[64]	SVG	1 game	Maxon RE40 12 V DC Motor, Apex Dynamics Gearbox Reduction, Autonics E40S6-5000–6-L-5 pulse rotary encoder, Arduino MEGA, Vnh2sp30 DC motor shield, security system	Unity	Functional: motor	Feedback (visual), Rules, Time, Rewards, Tasks, Speed	Game Mode (single-player), Simplicity, Adaptability (performance)	Points, Avatar, Aesthetic (2D, platforms)
[65]	Tangible SG	1 game	Robotic Glove, Microcontroller, Pressure sensors, LCD screen, Tactile Buttons	Not Aplicable	Functional: Motor, ADL	Help (assistance), Rules, Rewards, Tasks	Game Mode (single-player, accompanied by the therapist)	Points
[66]	SVG	1 game	A401 FSR, UDOO single board computer, security system	Unity	Functional: Motor (clinic)	Feedback (visual), Rules, Tasks	Game Mode (single-player), Simplicity	NPC (neutrals), Aesthetic (3D)
[67]	SVG	1 game	Myo ArmBand EMG controller, HMD system, PC	Unity, GUI	Functional: Motor (clinic)	Feedback (visual, sound, postgame), Rules, Rewards, Difficulties, Lifes	Game Mode (singleplayer, PvE), Progress	Points, NPC (hostile), Aesthetic (3D, sounds and music, first person)
[68]	SVG	7 Virtual Environments	PC	NeuroVR 2.0	Functional: ADL (clinic)	Feedback (visual, sound), Rules, Challenges, Difficulties	Game Mode (single-player, accompanied by the therapist)	NPC, Stories, Aesthetic (3D, first person)
[69]	IVR	4 games	Leap Motion Controller, HMD	Not Specified	Functional: Motor, ADL (clinic)	Feedback (visual), Rules, Rewards, Difficulty	Game Mode (single-player)	Points, Avatar, Levels, Aesthetic (3D, realistic and casual, music, first person)
[70]	IVR	1 game	HMD Oculus Rift CV1, Oculus Touch Controller	Unity, GUI	Functional: Motor (clinic)	Feedback (visual, sound, haptic), Rules, Rewards, Tasks	Game Mode (single-player), Story	Points, Levels, Avatar, Aesthetic (3D, first person), NPC (neutrals)
[71]	IVR	2 Virtual Environments	Google Cardboard, smartphone	Google Cardboard VR plug-in, Unity, Adobe Photoshop, Baidu AI Platform	Functional: Cognitive, ADL (clinic, telerehabilitation)	Feedback (visual), Rules, Tasks, Help (instructions)	Game Mode (single-player)	NPC, Aesthetic (3D, first person)
[72]	IVR	Virtual Environment	HMD Oculus Rift	3ds MAX (Autodesk), Unity	Functional: Motor (clinic)	Feedback (visual, postgame), Rules, Tasks, Time (spent)	Game Mode (single-player), Progress	Levels, Aesthetic (3D, music)

References

1. Sánchez-Gil, J.J., Sáez-Manzano, A., López-Luque, R., Ochoa-Sepúlveda, J.-J., Cañete-Carmona, E.: Gamified devices for stroke rehabilitation: A systematic review. Computer Methods and Programs in Biomedicine, 108476 (2024) https://doi.org/10.1016/j.cmpb.2024.108476
2. Burdea, G., Kim, N., Polistico, K., Kadaru, A., Grampurohit, N., Hundal, J., Pollack, S.: Robotic table and serious games for integrative rehabilitation in the early poststroke phase: Two case reports. JMIR Rehabilitation and Assistive Technologies **9**(2) (2022) https://doi.org/10.2196/26990
3. Moldoveanu, A., Ferche, O.-M., Moldoveanu, F., Lupu, R.G., Cinteză, D., Constantin Irimia, D., Toader, C.: The travee system for a multimodal neuromotor rehabilitation. IEEE Access **7**, 8151–8171 (2019) https://doi.org/10.1109/ACCESS.2018.2886271
4. Miao, Y., Chen, S., Zhang, X., Jin, J., Xu, R., Daly, I., Jia, J., Cichocki, A., Jung, T.-P.: Bci-based rehabilitation on the stroke in sequela stage. Neural Plasticity **2020**, 8882764 (2020) https://doi.org/10.1155/2020/8882764
5. Noveletto, F., Soares, A.V., Eichinger, F.L.F., Domenech, S.C., Hounsell, M.d.S., Filho, P.B.: Biomedical serious game system for lower limb motor rehabilitation of hemiparetic stroke patients. IEEE Transactions on Neural Systems and Rehabilitation Engineering **28**(6), 1481–1487 (2020) https://doi.org/10.1109/TNSRE.2020.2988362
6. Jung, H.-T., Daneault, J.-F., Lee, H., Kim, K., Kim, B., Park, S., Ryu, T., Kim, Y., Lee, S.I.: Remote assessment of cognitive impairment level based on serious mobile game performance: An initial proof of concept. IEEE Journal of Biomedical and Health Informatics **23**(3), 1269–1277 (2019) https://doi.org/10.1109/JBHI.2019.2893897
7. Song, X., Van De Ven, S.S., Liu, L., Wouda, F.J., Wang, H., Shull, P.B.: Activities of daily living-based rehabilitation system for arm and hand motor function retraining after stroke. IEEE Transactions on Neural Systems and Rehabilitation Engineering **30**, 621–631 (2022) https://doi.org/10.1109/TNSRE.2022.3156387
8. Feingold-Polak, R., Barzel, O., Levy-Tzedek, S.: A robot goes to rehab: a novel gamified system for long-term stroke rehabilitation using a socially assistive robot—methodology and usability testing. Journal of NeuroEngineering and Rehabilitation **18**(122) (2021) https://doi.org/10.1186/s12984-021-00915-2
9. Navarro, M.D., Llorens, R., Borrego, A., Alcañiz, M., Noé, E., Ferri, J.: Competition enhances the effectiveness and motivation of attention rehabilitation after stroke. a randomized controlled trial. Frontiers in Human Neuroscience **14** (2020) https://doi.org/10.3389/fnhum.2020.575403
10. Pouplin, S., Bonnyaud, C., Bouchigny, S., Mégard, C., Bertholier, L., Goulamhoussen, R., Foulon, P., Bensmail, D., Barbot, F., Roche, N.: Feasibility of a serious game system including a tangible object for post stroke upper limb rehabilitation: a pilot randomized clinical study. Front. Neurol. **14**, 1176071 (2023) https://doi.org/10.3389/fneur.2023.1176071
11. Pereira, F., Badia, S., Jorge, C., *et al.*: The use of game modes to promote engagement and social involvement in multi-user serious games: a within-person randomized trial with stroke survivors. Journal of NeuroEngineering and Rehabilitation **18**, 62 (2021) https://doi.org/10.1186/s12984-021-00853-z
12. Hung, J.-W., Chou, C.-X., Chang, Y.-J., Wu, C.-Y., Chang, K.-C., Wu, W.-C., Howell, S.: Comparison of kinect2scratch game-based training and therapist-based training for the improvement of upper extremity functions of patients with chronic stroke: a randomized controlled single-blinded trial. European Journal of Physical and Rehabilitation Medicine **55**(5), 542–550 (2019) https://doi.org/10.23736/S1973-9087.19.05598-9
13. Hung, N.T., Paul, V., Prakash, P., Kovach, T., Tacy, G., Tomic, G., Park, S., Jacobson, T., Jampol, A., Patel, P., Chappel, A., King, E., Slutzky, M.W.: Wearable myoelectric interface enables high-dose, home-based training in severely impaired chronic stroke survivors. Annals of Clinical and Translational Neurology **8**(9), 1895–1905 (2021) https://doi.org/10.1002/acn3.51442
14. Beauchamp, J.E.S., Wang, M., Leon Novelo, L.G., Cox, C., Meyer, T., Fagundes, C., Savitz, S.I., Sharrief, A., Dishman, D., Johnson, C.: Feasibility and user-experience of a virtual environment for social connection and education after stroke: A pilot study. Journal of Stroke and Cerebrovascular Diseases **33**(2), 107515 (2023) https://doi.org/10.1016/j.jstrokecerebrovasdis.2023.107515
15. Olafsdottir, S.A., Jonsdottir, H., Bjartmarz, I., *et al.*: Feasibility of activables to promote home-based exercise and physical activity of community-dwelling stroke survivors with support from caregivers: A mixed methods study. BMC Health Services Research **20**, 562 (2020) https://doi.org/10.1186/s12913-020-05432-x
16. Olafsdottir, S.A., Jonsdottir, H., Magnusson, C., al.: Developing activables for community-dwelling stroke survivors using the medical research council framework

for complex interventions. BMC Health Services Research **20**, 463 (2020) https://doi.org/10.1186/s12913-020-05198-2

17. Vourvopoulos, A., Marin-Pardo, O., Lefebvre, S., Neureither, M., Saldana, D., Jahng, E., Liew, S.-L.: Effects of a brain-computer interface with virtual reality (vr) neurofeedback: A pilot study in chronic stroke patients. Frontiers in Human Neuroscience **13** (2019) https://doi.org/10.3389/fnhum.2019.00210
18. Chatterjee, K., Buchanan, A., Cottrell, K., Hughes, S., Day, T.W., John, N.W.: Immersive virtual reality for the cognitive rehabilitation of stroke survivors. IEEE Transactions on Neural Systems and Rehabilitation Engineering **30**, 719–728 (2022) https://doi.org/10.1109/TNSRE.2022.3158731
19. Solanki, D., Lahiri, U.: Adaptive treadmill-assisted virtual reality-based gait rehabilitation for post-stroke physical reconditioning—a feasibility study in low-resource settings. IEEE Access **8**, 88830–88843 (2020) https://doi.org/10.1109/ACCESS.2020.2994081
20. Liu, L.Y., Sangani, S., Patterson, K.K., Fung, J., Lamontagne, A.: Real-time avatar-based feedback to enhance the symmetry of spatiotemporal parameters after stroke: Instantaneous effects of different avatar views. IEEE Transactions on Neural Systems and Rehabilitation Engineering **28**(4), 878–887 (2020) https://doi.org/10.1109/TNSRE.2020.2979830
21. Herne, R., Shiratuddin, M.F., Rai, S., Blacker, D., Laga, H.: Improving engagement of stroke survivors using desktop virtual reality-based serious games for upper limb rehabilitation: A multiple case study. IEEE Access **10**, 46354–46371 (2022) https://doi.org/10.1109/ACCESS.2022.3169286
22. Spreij, L.A., Visser-Meily, J.M.A., Sibbel, J., Gossett, I.K., Nijboer, T.C.W.: Feasibility and user-experience of virtual reality in neuropsychological assessment following stroke. Neuropsychological Rehabilitation **32**(4), 499–519 (2022) https://doi.org/10.1080/09602011.2020.1831935
23. Moan, M.E., Vonstad, E.K., Su, X., Vereijken, B., Solbjør, M., Skjæret-Maroni, N.: Experiences of stroke survivors and clinicians with a fully immersive virtual reality treadmill exergame for stroke rehabilitation: A qualitative pilot study. Frontiers in Aging Neuroscience **13** (2021) https://doi.org/10.3389/fnagi.2021.735251
24. Wu, J.-J., Zheng, M.-X., Hua, X.-Y., Wei, D., Xue, X., Lin, Y.-L., Xing, X.-X., Ma, J., Shan, C.-L., Xu, J.-G.: Altered effective connectivity in the emotional network induced by immersive virtual reality rehabilitation for post-stroke depression. Frontiers in Human Neuroscience **16** (2022) https://doi.org/10.3389/fnhum.2022.974393
25. Aguilera-Rubio, , Cuesta-Gómez, A., Mallo-López, A., Jardón-Huete, A., Oña-Simbaña, E.D., Alguacil-Diego, I.M.: Feasibility and efficacy of a virtual reality game-based upper extremity motor function rehabilitation therapy in patients with chronic stroke: A pilot study. International Journal of Environmental Research and Public Health **19**, 3381 (2022) https://doi.org/10.3390/ijerph19063381
26. Marques, I.A., Alves, C.M., Rezende, A.R., Mendes, L.C., Paiva, T.S., Cyrino, G.F., Souza, J.T., Silva, M.A.M., Souza, L.A.P.S., Naves, E.L.M.: Virtual reality and serious game therapy for post-stroke individuals: A preliminary study with humanized rehabilitation approach protocol. Complementary Therapies in Clinical Practice **49**, 101681 (2022) https://doi.org/10.1016/j.ctcp.2022.101681
27. Shahmoradi, L., Almasi, S., Ahmadi, H., Bashiri, A., Azadi, T., Mirbagherie, A., Ansari, N.N., Honarpishe, R.: Virtual reality games for rehabilitation of upper extremities in stroke patients. Journal of Bodywork and Movement Therapies **26**, 113–122 (2021) https://doi.org/10.1016/j.jbmt.2020.10.006
28. Song, Z., Fan, X., Dong, J., Zhang, X., Xu, X., Li, W., Pu, F.: The third-person perspective full-body illusion induced by visual-tactile stimulation in virtual reality for stroke patients. Consciousness and Cognition **115**, 103578 (2023) https://doi.org/10.1016/j.concog.2023.103578
29. Specht, J., Schroeder, H., Krakow, K., Meinhardt, G., Stegmann, B., Meinhardt-Injac, B.: Acceptance of immersive head-mounted display virtual reality in stroke patients. Computers in Human Behavior Reports **4**, 100141 (2021) https://doi.org/10.1016/j.chbr.2021.100141
30. Thompson-Butel, A.G., Shiner, C.T., McGhee, J., Bailey, B.J., Bou-Haidar, P., McCorriston, M., Faux, S.G.: The role of personalized virtual reality in education for patients post stroke—a qualitative case series. Journal of Stroke and Cerebrovascular Diseases **28**(2), 450–457 (2019) https://doi.org/10.1016/j.jstrokecerebrovasdis.2018.10.018
31. Dong, Y., Liu, X., Tang, M., *et al.*: A haptic-feedback virtual reality system to improve the box and block test (bbt) for upper extremity motor function assessment. Virtual Reality **27**, 1199–1219 (2023) https://doi.org/10.1007/s10055-022-00727-2
32. Fu, M.J., Harley, M.Y., Hisel, T., Busch, R., Wilson, R., Chae, J., Knutson, J.S.: Ability of people with post-stroke hemiplegia to self-administer fes-assisted hand therapy video games at home: An exploratory case series. Journal of Rehabilitation and Assistive Technologies Engineering **6** (2019) https://doi.org/

10.1177/2055668319854000 . PMID: 31360537
33. Tannous, H., Istrate, D., Perrochon, A., Daviet, J.-C., Benlarbi-Delai, A., Sarrazin, J., Ho Ba Tho, M.-C., Dao, T.T.: Gamerehab@home: A new engineering system using serious game and multisensor fusion for functional rehabilitation at home. IEEE Transactions on Games **13**(1), 89–98 (2021)
34. Caggianese, G., Cuomo, S., Esposito, M., Franceschini, M., Gallo, L., Infarinato, F., Minutolo, A., Piccialli, F., Romano, P.: Serious games and in-cloud data analytics for the virtualization and personalization of rehabilitation treatments. IEEE Transactions on Industrial Informatics **15**(1), 517–526 (2019) https://doi.org/10.1109/TII.2018.2856097
35. Rivas, J.J., Orihuela-Espina, F., Palafox, L., Bianchi-Berthouze, N., Lara, M.d.C., Hernández-Franco, J., Sucar, L.E.: Unobtrusive inference of affective states in virtual rehabilitation from upper limb motions: A feasibility study. IEEE Transactions on Affective Computing **11**(3), 470–481 (2020) https://doi.org/10.1109/TAFFC.2018.2808295
36. Rivas, J.J., Carmen Lara, M., Castrejón, L., Hernández-Franco, J., Orihuela-Espina, F., Palafox, L., Williams, A., Bianchi-Berthouze, N., Sucar, L.E.: Multi-label and multimodal classifier for affective states recognition in virtual rehabilitation. IEEE Transactions on Affective Computing **13**(3), 1183–1194 (2022) https://doi.org/10.1109/TAFFC.2021.3055790
37. Cheng, N., Phua, K.S., Lai, H.S., Tam, P.K., Tang, K.Y., Cheng, K.K., Yeow, R.C.-H., Ang, K.K., Guan, C., Lim, J.H.: Brain-computer interface-based soft robotic glove rehabilitation for stroke. IEEE Transactions on Biomedical Engineering **67**(12), 3339–3351 (2020) https://doi.org/10.1109/TBME.2020.2984003
38. Bai, J., Song, A.: Development of a novel home based multi-scene upper limb rehabilitation training and evaluation system for post-stroke patients. IEEE Access **7**, 9667–9677 (2019) https://doi.org/10.1109/ACCESS.2019.2891606
39. Dash, A., Lahiri, U.: Design of virtual reality-enabled surface electromyogram-triggered grip exercise platform. IEEE Transactions on Neural Systems and Rehabilitation Engineering **28**(2), 444–452 (2020) https://doi.org/10.1109/TNSRE.2019.2959449
40. Bae, S., Park, H.-S.: Development of immersive virtual reality-based hand rehabilitation system using a gesture-controlled rhythm game with vibrotactile feedback: An fnirs pilot study. IEEE Transactions on Neural Systems and Rehabilitation Engineering **31**, 3732–3743 (2023) https://doi.org/10.1109/TNSRE.2023.3312336
41. He, C., Xiong, C.-H., Chen, Z.-J., Fan, W., Huang, X.-L., Fu, C.: Preliminary assessment of a postural synergy-based exoskeleton for post-stroke upper limb rehabilitation. IEEE Transactions on Neural Systems and Rehabilitation Engineering **29**, 1795–1805 (2021) https://doi.org/10.1109/TNSRE.2021.3107376
42. Hougaard, B.I., Knoche, H., Kristensen, M.S., Jochumsen, M.: Modulating frustration and agency using fabricated input for motor imagery bcis in stroke rehabilitation. IEEE Access **10**, 72312–72327 (2022) https://doi.org/10.1109/ACCESS.2022.3188103
43. Crema, A., Furfaro, I., Raschellà, F., Rossini, M., Zajc, J., Wiesener, C., Baccinelli, W., Proserpio, D., Augusten, A., Immick, N., Becker, S., Weber, M., Schauer, T., Krakow, K., Gasperini, G., Molteni, F., Russold, M.F., Bulgheroni, M., Micera, S.: Reactive exercises with interactive objects: Interim analysis of a randomized trial on task-driven nmes grasp rehabilitation for subacute and early chronic stroke patients. Sensors **21**, 6739 (2021) https://doi.org/10.3390/s21206739
44. Wu, J., Dodakian, L., See, J., Burke Quinlan, E., Meng, L., Abraham, J., Wong, E.C., Le, V., McKenzie, A., Cramer, S.C.: Gains across who dimensions of function after robot-based therapy in stroke subjects. Neurorehabil Neural Repair **34**(12), 1150–1158 (2020) https://doi.org/10.1177/1545968320956648 . Epub 2020 Oct 21
45. Faria, A.L., Pinho, M.S., Badia, S.: A comparison of two personalization and adaptive cognitive rehabilitation approaches: a randomized controlled trial with chronic stroke patients. Journal of NeuroEngineering and Rehabilitation **17**, 78 (2020) https://doi.org/10.1186/s12984-020-00691-5
46. Heinrich, C., Morkisch, N., Langlotz, T., *et al.*: Feasibility and psychophysical effects of immersive virtual reality-based mirror therapy. Journal of NeuroEngineering and Rehabilitation **19**, 107 (2022) https://doi.org/10.1186/s12984-022-01086-4
47. Thielbar, K., Triandafilou, K., Barry, A., Yuan, N., Nishimoto, A., Johnson, J., Stoykov, M., Tsoupikova, D., Kamper, D.: Home-based upper extremity stroke therapy using a multiuser virtual reality environment: A randomized trial. Archives of Physical Medicine and Rehabilitation **101** (2019) https://doi.org/10.1016/j.apmr.2019.10.182
48. Winter, C., Kern, F., Gall, D., et al.: Immersive virtual reality during gait rehabilitation increases walking speed and motivation: a usability evaluation with healthy participants and patients with multiple sclerosis and stroke. Journal of NeuroEngineering and Rehabilitation **18**(68) (2021) https://doi.org/10.1186/s12984-021-00848-w
49. Bobin, M., Bimbard, F., Boukallel, M., Anastassova,

M., Ammi, M.: Spectrum: Smart ecosystem for stroke patients upper limbs monitoring. Smart Health **13**, 100066 (2019) https://doi.org/10.1016/j.smhl.2019.01.001
50. Bhatia, D., Jo, S.H., Ryu, Y., Kim, Y., Kim, D.H., Park, H.-S.: Wearable triboelectric nanogenerator based exercise system for upper limb rehabilitation post neurological injuries. Nano Energy **80**, 105508 (2021) https://doi.org/10.1016/j.nanoen.2020.105508
51. Wagner, S., Belger, J., Joeres, F., Thöne-Otto, A., Hansen, C., Preim, B., Saalfeld, P.: ivroad: Immersive virtual road crossing as an assessment tool for unilateral spatial neglect. Computers Graphics **99**, 70–82 (2021) https://doi.org/10.1016/j.cag.2021.06.013
52. Bakker, M.D.J., Boonstra, N., Nijboer, T.C.W., Holstege, M.S., Achterberg, W.P., Chavannes, N.H.: The design choices for the development of an augmented reality game for people with visuospatial neglect. Clinical eHealth **3**, 82–88 (2020) https://doi.org/10.1016/j.ceh.2020.11.003
53. Alhirsan, S.M., Capó-Lugo, C.E., Hurt, C.P., Uswatte, G., Qu, H., Brown, D.A.: The immediate effects of different types of augmented feedback on fast walking speed performance and intrinsic motivation after stroke. Archives of Rehabilitation Research and Clinical Translation **5**(2), 100265 (2023) https://doi.org/10.1016/j.arrct.2023.100265
54. Bouatrous, A., Meziane, A., Zenati, N., *et al.*: A new adaptive vr-based exergame for hand rehabilitation after stroke. Multimedia Systems **29**, 3385–3402 (2023) https://doi.org/10.1007/s00530-023-01180-0
55. Everard, G., Otmane-Tolba, Y., Rosselli, Z., et al.: Concurrent validity of an immersive virtual reality version of the box and block test to assess manual dexterity among patients with stroke. Journal of NeuroEngineering and Rehabilitation **19**(7) (2022) https://doi.org/10.1186/s12984-022-00981-0
56. Høeg, E.R., Bruun-Pedersen, J.R., Cheary, S., *et al.*: Buddy biking: a user study on social collaboration in a virtual reality exergame for rehabilitation. Virtual Reality **27**, 245–262 (2023) https://doi.org/10.1007/s10055-021-00544-z
57. Cha, K., Wang, J., Li, Y., et al.: A novel upper-limb tracking system in a virtual environment for stroke rehabilitation. Journal of NeuroEngineering and Rehabilitation **18**(166) (2021) https://doi.org/10.1186/s12984-021-00957-6
58. Hogan, C., Cornwell, P., Fleming, J., *et al.*: Assessment of prospective memory after stroke utilizing virtual reality. Virtual Reality **27**, 333–346 (2023) https://doi.org/10.1007/s10055-021-00576-5
59. Painter, D.R., Norwood, M.F., Marsh, C.H., et al.: Immersive virtual reality gameplay detects visuospatial atypicality, including unilateral spatial neglect, following brain injury: a pilot study. Journal of NeuroEngineering and Rehabilitation **20**(161) (2023) https://doi.org/10.1186/s12984-023-01283-9
60. Oliveira, J., Gamito, P., Pereira, R., *et al.*: Virtual and real atm use performance in patients with acquired brain injury and healthy controls. Virtual Reality **27**, 2431–2440 (2023) https://doi.org/10.1007/s10055-023-00819-7
61. Knippenberg, E., Timmermans, A., Coolen, J., et al.: Efficacy of a technology-based client-centred training system in neurological rehabilitation: a randomised controlled trial. Journal of NeuroEngineering and Rehabilitation **18**(184) (2021) https://doi.org/10.1186/s12984-021-00977-2
62. Alves, T., Gonçalves, R.S., Carbone, G.: Serious games strategies with cable-driven robots for bimanual rehabilitation: A randomized controlled trial with post-stroke patients. Frontiers in Robotics and AI **9** (2022) https://doi.org/10.3389/frobt.2022.739088
63. Alves, T., Gonçalves, R.S., Carbone, G.: Quantitative progress evaluation of post-stroke patients using a novel bimanual cable-driven robot. Journal of Bionic Engineering **18**, 1331–1343 (2021) https://doi.org/10.1007/s42235-021-00102-y
64. Rodrigues, L.A.O., Moraes, V.P., Gonçalves, R.S.: Regear: an upper and lower limb simultaneous system for stroke rehabilitation. Journal of the Brazilian Society of Mechanical Sciences and Engineering **43**, 488 (2021) https://doi.org/10.1007/s40430-021-03199-8
65. Lim, D.Y., Lai, H., Yeow, R.C.: A bidirectional fabric-based soft robotic glove for hand function assistance in patients with chronic stroke. Journal of NeuroEngineering and Rehabilitation **20**(120) (2023) https://doi.org/10.1186/s12984-023-01250-4
66. Thijs, L., Voets, E., Wiskerke, E., et al.: Technology-supported sitting balance therapy versus usual care in the chronic stage after stroke: a pilot randomized controlled trial. Journal of NeuroEngineering and Rehabilitation **18**(120) (2021) https://doi.org/10.1186/s12984-021-00910-7
67. Montoya, M.F., Muñoz, J., Henao, O.A.: Fatigue-aware videogame using biocybernetic adaptation: a pilot study for upper-limb rehabilitation with semg. Virtual Reality **27**, 277–290 (2023) https://doi.org/10.1007/s10055-021-00561-y
68. Giachero, A., Calati, M., Pia, L., Vista, L.L., Molo, M., Rugiero, C., Fornaro, C., Fornaro, C.: Conversational therapy through semi-immersive virtual reality environments for language recovery and psychological wellbeing in post stroke aphasia. Behavioural Neurology, 15

(2020). Article ID 2846046
69. ÖGÜN, M.N., KURUL, R., YAŞAR, M.F., TURKOGLU, S.A., AVCI, YILDIZ, N.: Effect of leap motion-based 3d immersive virtual reality usage on upper extremity function in ischemic stroke patients. Arquivos de Neuro-Psiquiatria **77**(10) (2019)
70. Huygelier, H., Schraepen, B., Lafosse, C., Vaes, N., Schillebeeckx, F., Michiels, K., Note, E., Vanden Abeele, V., Ee, R., Gillebert, C.R.: An immersive virtual reality game to train spatial attention orientation after stroke: A feasibility study. Applied Neuropsychology: Adult **29**(5), 915–935 (2022) https://doi.org/10.1080/23279095.2020.1821030 2020 Sep 18
71. Bu, X., Ng, P., Tong, Y., Chen, P., Fan, R., Tang, Q., Cheng, Q., Li, S., Cheng, A., Liu, X.: A mobile-based virtual reality speech rehabilitation app for patients with aphasia after stroke: Development and pilot usability study. JMIR Serious Games **10**(2), 30196 (2022) https://doi.org/10.2196/30196
72. Iosa, M., Aydin, M., Candelise, C., Coda, N., Morone, G., Antonucci, G., Marinozzi, F., Bini, F., Paolucci, S., Tieri, G.: The michelangelo effect: Art improves the performance in a virtual reality task developed for upper limb neurorehabilitation. Frontiers in Psychology **11**, 611956 (2021) https://doi.org/10.3389/fpsyg.2020.611956
